# Does humidity matter? Prenatal heat and child health in South Asia

**DOI:** 10.1126/sciadv.adx3010

**Published:** 2025-12-19

**Authors:** Kathryn McMahon, Kathy Baylis, Stuart Sweeney, Chris Funk

**Affiliations:** ^1^ Department of Geography, University of California Santa Barbara, Santa Barbara, CA 93106, USA.; ^2^ Climate Hazards Center, University of California Santa Barbara, Santa Barbara, CA 93106, USA.

## Abstract

Heat extremes pose substantial health risks during pregnancy and early childhood. High humidity exacerbates heat strain, but its long-term effects on health remain poorly understood. We compare the effect of prenatal exposure to extreme humid heat versus heat alone on child growth in South Asia, where high rates of child stunting meet rapidly accelerating hot-humid extremes. After adjusting for sociodemographic, seasonal, and spatial confounders, we use within-community variation in children’s ages to isolate the impact of prenatal exposures. We find that hot-humid exposures are much more detrimental to health than hot temperatures alone, with the potential to increase stunting in South Asia by over 3 million children by 2050. These findings underscore the importance of accounting for humidity when estimating and localizing climate change impacts.

## Teaser

Prenatal humid heat extremes undermine child health in South Asia, reducing growth more severely than hot temperatures alone.

## INTRODUCTION

Because of climate change, extreme heat events are increasing in frequency, intensity, and duration, posing an immediate and growing threat to human health (*1*). While the scientific literature has largely focused on the important issue of heat-related mortality (*2*–*4*), the lasting health impacts of heat on the living are often overlooked, despite growing evidence linking extremes with higher morbidity (*5*–*8*). Even in healthy adults, prolonged exposure to hot environments can raise body temperatures to dangerous levels, straining the heart and increasing rates of both temporary and permanent organ damage, particularly for the kidneys (*5*–*7*). Physiologically and socially vulnerable groups suffer even higher rates of extreme heat exposure and associated adverse outcomes. Among those most vulnerable are the very young and the very old, pregnant people, poor and marginalized populations, and those engaged in strenuous outdoor labor (*9*–*13*).

Extensive evidence from physiology shows that high humidity exacerbates heat strain by preventing the evaporation of sweat from our skin, inhibiting the body’s natural cooling mechanism (*14*–*17*). This body of work suggests that ambient air temperature is, at best, a coarse measure of the biological stress associated with heat exposure, which is better captured by composite metrics like the wet-bulb globe temperature that account for the additional factors—particularly humidity—that lead to heat stress (*18*–*20*). However, these results conflict with numerous epidemiological studies which find that humidity has little or no moderating effect on estimated relationships between temperature and rates of mortality or acute morbidity using observational data (*5*, *21*–*24*). Meanwhile, the broader scientific community continues to focus primarily on the risks associated with extreme temperatures alone. Reconciling these conflicting findings is critical for evaluating and addressing the health costs of climate change (*25*). If true additional health risks of high humidity are present but missed, studies that focus exclusively on temperature and exposure assessments will underestimate the real cost of climate change for health, especially in the hot and humid global tropics (*26*). Given that humid areas—such as river valleys and coastlines—are often densely populated, such studies may also critically misrepresent the total population at risk and their location.

Pregnant women and their babies are at a heightened risk for the health consequences associated with hot and hot-humid environments. One multinational study in sub-Saharan Africa finds that mean birth weights fall by up to 0.9 g for each day during pregnancy where temperatures reach 100°F (38°C) (*27*), while another finds that a 10% increase in the number of days over 104°F (40°C) raises the likelihood of late-stage pregnancy loss by 1.9% (*28*). Given recent advancements in satellite-enhanced gridded temperature data (*29*, *30*) and an increasing understanding of the importance of additional environmental factors like humidity (*31*), these studies may underestimate the true effect of extreme heat.

At least three key biological mechanisms interact to heighten the dangers of heat exposure for pregnant women and infants. Two of these are changing hormone levels and increased metabolic heat production during pregnancy, which both inhibit natural cooling and make maternal core temperature more sensitive to the effects of heat exposure. Third, dehydration during heat events has been shown to induce labor early in some women (*32*). Together, these factors increase the risk of maternal heat stress, pregnancy loss, stillbirth, preterm birth, and low birth weights (*13*, *28*, *33*–*38*), but they may be sensitive to differences across the temperature-humidity gradient. For example, research from physiology suggests that core temperature may be most responsive to hot-humid environments, while dehydration-related preterm birth may be more common under hot-dry conditions due to elevated rates of sweat loss.

By undermining infant health at birth, prenatal heat exposures also threaten long-term health and well-being. Low health markers at birth—which are more common among babies who are born prematurely or at term but with low birth weight—are consistently associated with adverse outcomes as a child grows (*39*–*42*). External shocks to health and nutrition during the prenatal period have been quantifiably linked to educational, financial, and physical impacts well into adulthood (*43*–*46*). The child height-for-age ratio is a commonly used indicator of chronic health status for children under the age of five and therefore constitutes a useful landmark connecting prenatal exposures, infant health, and cascading effects into adulthood (*37*, *47*, *48*). If hot and hot-humid extremes have different effects on height-for-age, then these differences are likely to translate into lasting downstream consequences for health and economic well-being throughout the life course.

Social and physical vulnerability to extreme heat intersect in South Asia, where inequalities in resource access and high rates of child undernutrition come head-to-head with rapidly accelerating exposure to extreme heat and humidity (*49*, *50*). Now and in the future, hot-humid conditions are concentrated along South Asia’s river valleys and coasts, which are also home to some of the densest populations in the world [fig. S3; (*51*)]. Even if societies succeed in limiting warming to 2°C above preindustrial levels, South Asia is expected to suffer from deadly heat events every year (*52*). At the same time, rates of child stunting remain high throughout the region. In 2023, the United Nation’s Children’s Fund reported that South Asia was home to one third (54 million) of the world’s stunted children (*53*). Existing research shows that the burdens of child undernutrition and climate change are inextricably linked. Nutritional status is highly sensitive to environmental shocks up to age 5 (*54*), particularly for children in poor households who often lack the social and material resources necessary for shock mitigation and recovery (*48*, *55*).

Here, we conduct a fine-scale (10 km) analysis of the effects of prenatal exposure to extreme heat and humidity on height attainment for approximately 200,000 children in Bangladesh, India, and Nepal. After controlling for a comprehensive set of demographic characteristics and spatio-temporal confounders related to nutrition, we use short-run variation in daily maximum temperature (T_max_) and maximum wet-bulb globe temperature (WBGT_max_) in the trimesters before birth to observe the effect of heat extremes on height-for-age *z*-scores (HAZ) in children under the age of five, a key indicator of chronic undernutrition at a critical stage of growth and development. Our design adjusts for the nonrandom assignment of heat exposure across our sample from spatial and temporal factors using community-level fixed effects and state-by-survey year fixed effects. We also control for children’s calendar month of birth to account for seasonal dynamics. We therefore compare children with similar sociodemographic characteristics who were born in the same community and calendar month but in different years, which provides plausibly exogenous variation in the weather conditions during their prenatal periods. In a supplemental analysis, we further strengthen our identification of exogeneity by directly comparing children within the same household using household fixed effects.

When we define extreme heat using WBGT_max_, a heat stress metric that incorporates humidity, we find that prenatal heat exposure is more detrimental to child growth than when we estimate this relationship using temperature alone. Specifically, a one standard deviation increase in the number of hot-humid days in the third trimester decreases height-for-age by 5.1%. The corresponding decrease for just hot days would be 1.3%. Combined with new heat projections, our coefficient estimates on WBGT_max_ exposure imply that 3 to 3.7 million additional children would have been stunted across our study region had they been exposed to the levels of heat and humidity that are expected by 2050 under a high-emission climate change scenario. This estimate shrinks to 300,000 to 400,000 additional children when we apply the coefficient estimates on maximum temperature. We find that the main effects are robust across numerous alternative versions of the analysis that use absolute and relative heat thresholds, adjust explicitly for local historical climate averages, and directly compare siblings using household fixed effects.

This study evaluates the relative impacts of hot versus hot-humid prenatal environments on child growth trajectories at the multinational level. While earlier literature finds substantial effects of heat on mortality and explores the role of humidity in the heat-health relationship with mixed results, there is a lack of research exploring the effects of heat and humidity on long-term morbidity and its associated economic outcomes (*8*). Extreme heat harms many more people than it kills, and these lingering impacts have so far received little attention. Our findings shed light on this relationship, providing notable evidence that humid heat events pose a large and growing threat to long-term health and economic stability for children in the global tropics.

## RESULTS

### Heat exposure

**Fig. 1.**
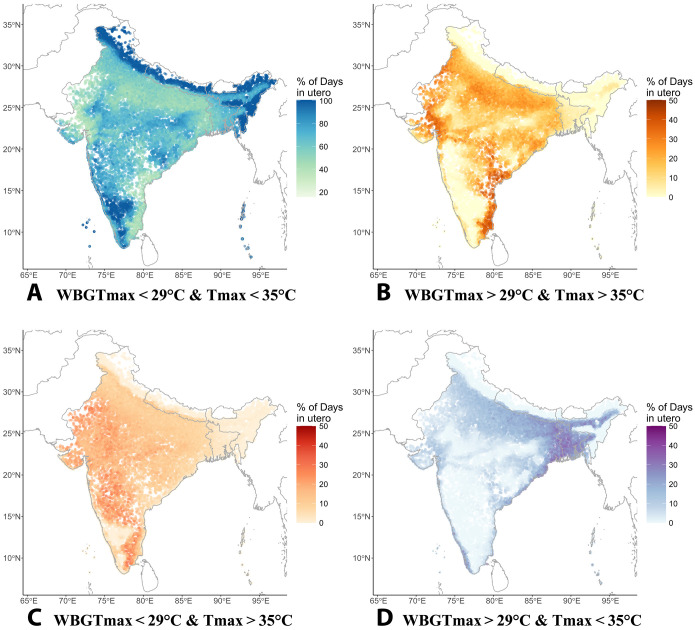
Spatial distribution of prenatal heat exposure in South Asia. Each point represents a cluster (*N* = 29,357) from the DHS. Point color is determined by the average number of days in each heat category experienced during trimesters 1 to 3 by 0-to-5 year olds in each cluster. (**A**) Percentage of total days that exceeded neither heat threshold. (**B**) Percentage of days that were extreme by both definitions. (**C**) Percentage of days with maximum temperatures (T_max_) above 35°C but maximum wet-bulb globe temperatures (WBGT_max_) below 29°C. (**D**) Percentage with WBGT_max_ > 29°C but T_max_ < 35°C.

Figure 1 describes the average conditions during the 9 months before birth for children in each community surveyed by the Demographic and Health Surveys (DHS). Each point represents a DHS survey location, or “cluster,” which is approximately the size of a rural village or urban city block. The color of the points is determined by each cluster’s average degree of exposure to our primary thresholds of maximum temperature (T_max_ > 35°C) and maximum wet-bulb globe temperature (WBGT_max_ > 29°C). These thresholds were selected using a quantile-based approach to ensure that extreme days under each definition occur with nearly equal frequency in our sample. Further discussion of the threshold choice and sensitivity checks using controls for historical heat exposure and locally defined relative thresholds can be found in the “Robustness checks” section and the Supplementary Materials. Figure 1A shows the average percentage of total days during pregnancy that were relatively cool and dry, exceeding neither heat threshold; Fig. 1B shows the corresponding percentage of days that exceeded both thresholds; Fig. 1C shows the percent of days where T_max_ exceeded 35°C but WBGT_max_ stayed below 29°C, indicating dry heat; and Fig. 1D shows the percent of days with WBGT_max_ > 29°C but T_max_ < 35°C, indicating warm temperatures with high humidity.

Figure 1 reveals that children born in Central Southern India and the high-elevation regions in Nepal and Northern and Eastern India experienced relatively cool and dry conditions in utero. On the other hand, children along the Southeast coast of India and the Northwest border with Pakistan—historically hot areas—were frequently exposed to days that were extremely hot by both definitions. Days in this most extreme category comprised up to 50% of the average pregnancy (or 135 to 140 days) in some survey locations. Last, while hot and dry days are most common in the central swaths of the region (Fig. 1C), those exceeding only our WBGT_max_ threshold (Fig. 1D) are more concentrated along the coast of the subcontinent where humidity is relatively high, as well as in the Indian foothills of the Himalayas and much of Bangladesh. These low, humid areas along the Ganges and Brahmaputra rivers are home to very dense populations, thus amplifying human exposure to humid heat extremes (fig. S3).

The differences in spatial distribution between Fig. 1 (C and D) suggest considerable variation in the subpopulations that are exposed to hot versus hot-humid heat. Our subsequent analyses explore this variation further by quantifying the relationship between child health and exposure to both heat extremes. The frequency of extreme days is largely consistent between T_max_ and WBGT_max_, and the distributions of each exhibit notable left skewness. See table S1 for summary statistics on heat exposure across trimesters and heat variables. Additional exploratory analyses reveal that there is a high positive correlation (Pearson coefficient = 0.93) between mean monthly T_max_ and WBGT_max_ during 1993 to 2016, which is the period in which our sample of children experienced their prenatal periods. The correlation becomes somewhat weaker in the right-hand tails of the distributions, resulting in a moderate positive correlation between the number of days with T_max_ > 35°C and WBGT_max_ > 29°C (Pearson coefficient = 0.67) at the trimester level. See fig. S20 for a scatterplot of mean monthly T_max_ versus WBGT_max_ at the cluster level during 1993 to 2016.

### Effects on height-for-age

**Fig. 2.**
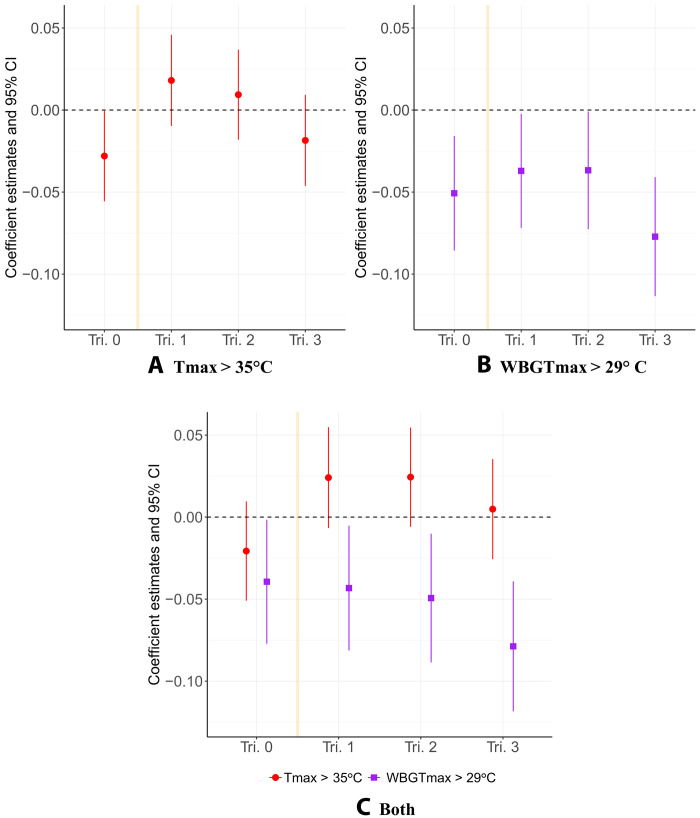
Main effects of heat exposure on HAZ. Coefficients and 95% confidence intervals (CI) for the effect of a one standard deviation change in the number of days with maximum temperature above 35°C (**A**), maximum wet-bulb globe temperature above 29°C (**B**), and all heat (**C**) on HAZ. “Tri. 0” refers to the three-month period before conception, which is represented in each plot with a yellow vertical line. Controls for child’s sex, twin status, birth order, birth location, child’s age in months, birth month, month of survey, mother’s age in years, mother’s educational attainment, parity, religion, marital status, and improved toilet access are included in the model but not shown. Fixed effects for cluster and state-by-survey-year are also omitted (see tables S2 to S6 for regression results).

Figure 2 presents the coefficients and 95% confidence intervals on prenatal heat exposure from our main models of HAZ, estimated using a comprehensive suite of fixed effects and demographic controls (see tables S2 to S6 for full regression results). Figure 2 (A and B) shows the coefficients on heat exposure estimated from our T_max_-only and WBGT_max_-only models, respectively, whereas Fig. 2C presents the results from our joint model with both metrics included. For ease of interoperability, we transform all heat exposure variables as a *z*-score of the number of extreme days per trimester. The coefficients may therefore be interpreted as the expected change in HAZ given a one standard deviation increase in the number of days with T_max_ > 35°C or WBGT_max_ > 29°C, depending on the model. In each case, these standardized variables for heat exposure from every trimester (0 to 3) are included among the covariates. See tables S3 to S7 for regression results using the nontransformed variables.

Figure 2A shows that maximum temperatures above 35°C are associated with statistically significant reductions in height-for-age when exposure occurs during the 3 months before conception (or “trimester 0”). We estimate that a one standard deviation increase in the number of days with T_max_ > 35°C during this preconception window decreases the conditional mean of HAZ by 0.03. This trimester 0 effect points to a little-documented but plausible mechanism related to maternal health at the outset of pregnancy (*27*). Meanwhile, heat exposure during trimesters 1, 2, and 3 have no statistically measurable effect on HAZ, although the negative coefficient on third-trimester exposures aligns with previous studies that find late-term heat exposure to be predictive of increased risk of adverse birth outcomes such as preterm birth and low birth weight (*28*, *37*).

Like T_max_, WBGT_max_ extremes are associated with lower HAZ in early childhood, particularly when exposure occurs at the beginning or the end of the prenatal year (Fig. 2B). Unlike T_max_, however, hot-humid heat during any trimester is detrimental to child health; all estimated coefficients are negative and statistically different from zero at the 5% level. Moreover, the negative effects of WBGT_max_ > 29°C on height attainment are considerably larger in magnitude than those of T_max_ > 35°C. Whereas a one standard deviation increase in days with T_max_ > 35°C during trimester 3 would suggest a reduction in HAZ by only 1.3% from the sample average (−1.57), an equivalent increase in days with WBGT_max_ > 29°C would imply a 5.1% decrease in HAZ. Our results further imply that heat exposure effects accumulate across trimesters. For example, we estimate that a child who experienced a one standard deviation increase in hot-humid days in every trimester would be 13% shorter for their age, relative to the sample mean, than a child with average exposure. For T_max_ > 35°C, the equivalent additive effect across trimesters would imply only a 1% decrease in HAZ. Note that the underlying biological mechanism may be more severe than this—for example, repeated exposure over multiple trimesters may accumulate multiplicatively rather than additively as our model specification suggests. Hence, a different design may be required to comprehensively assess the impacts of repeated exposures.

Last, we present coefficients from a third model (Fig. 2C) that allows for a more direct comparison of the relative health risks of hot versus hot-humid heat by including all T_max_ and WBGT_max_ exposure variables among the covariates. The results from this model reaffirm that days with WBGT_max_ > 29°C pose a greater threat to child health than days with T_max_ > 35°C. After controlling for hot-humid days, exposure to T_max_ > 35°C has no statistically detectable (α = 0.05) effect on child growth. Meanwhile, all coefficients on WBGT_max_ remain negative and statistically different from zero, and the confidence intervals on the estimated effect of T_max_ and WBGT_max_ exposures are now nonoverlapping in the second and third trimesters. While some of the polarization in effect sign may be attributable to the moderate positive correlation (Pearson coefficient = 0.67) between our two heat thresholds, it is notable that the effects of hot-humid extremes remain remarkably consistent with the WBGT_max_-only model (Fig. 2B).

To further characterize our main findings, we combine the effects presented in Fig. 2 (A and B) with new projections of WBGT_max_ and T_max_ produced by the Climate Hazards Center (*56*). These data leverage the same daily 0.05° heat records used in our main analysis, but they are now perturbed according to two 2050 climate change scenarios. The first uses the 2-4.5 Shared Socioeconomic Pathway (SSP), which represents a middle-of-the-road emissions scenario, and the second uses SSP5-8.5, a high-emission climate change scenario. We link our sample of children to the conditions they would have experienced during their prenatal year under both scenarios using DHS cluster-level geocodes (table S1). Because we use each child’s unique month, year, and location of birth to identify exposure to extremes, we retain full spatial and temporal variation in the projected data. With the resulting dataset, we calculate the difference between the projected and observed number of extreme days for each trimester for each child using each SSP scenario. Under the 2050 warming with SSP2-4.5, the average child in our sample is projected to experience approximately 31 days per trimester with WBGT_max_ > 29°C and 25 days with T_max_ > 35°C, representing a 47.6 and 31.6% increase in average exposure from our sample mean of 21 and 19 days, respectively. Under SSP5-8.5, exposures increase to 34 days per trimester with WBGT_max_ > 29°C and 27 days with T_max_ > 35°C on average, which implies exposures 61.9 and 42.1% higher than those in the observed data. Note that, in both cases, the rate of increase in exposure is projected to be greater for humid heat.

Next, for each scenario, we multiply the projected change in extreme days for each child under each SSP scenario (table S1) by the coefficients on WBGT_max_ > 29°C and T_max_ > 35°C from a version of our main models without the *z*-score transformation on extreme heat (tables S3 to S7) and then sum the results across all trimesters. We use the nonstandardized heat exposure variables here so that the coefficients represent the estimated mean effect of adding one extreme day per trimester and can therefore be multiplied by the projected change in extreme days and summed to recover the estimated effect of rising exposure on HAZ. From the WBGT_max_ model, we calculate that an additional 0.02 to 0.03% (*N* = 4543 to *N* = 5628) of all children in our dataset would be stunted had their prenatal period occurred under 2050 conditions under SSP2-4.5 and SSP5-8.5, respectively. After applying DHS survey weights to obtain representative estimates, this fraction contains between 3 and 3.7 million children depending on the scenario. These weights are derived using the United Nations Population Division population estimates from each county at the time of each DHS survey round (*57*).

Using the same logic and corresponding point estimates from our regression of HAZ on T_max_ exposure (Fig. 2A), we calculate that elevated temperatures under 2050 warming would only have the power to increase total stunting by an additional 300,000 to 400,000 children relative to the DHS sample. This difference implies that failing to account for the added effect of humidity would lead us to underestimate the vulnerable population by between 2.7 and 3.3 million children. Note that these estimates carry a large degree of uncertainty from several sources, including our coefficient estimates, overall statistical model, and projections of temperature and wet-bulb globe temperature. They should be understood as an imperfect illustration of the relative magnitude of our coefficients on WBGT_max_ and T_max_ rather than a precise prediction of stunting rates in 2050. Last, given that population growth is often the largest contributor to rising rates of hot-humid heat exposure in South Asia (*50*), this approximation may be an underestimate of the true number of children who will be vulnerable to heat-induced stunting in 2050 after accounting for the growing population.

### Robustness checks

We run a number of additional model specifications to alleviate concerns regarding causality in our main results. First, because the results presented in Fig. 2 only include children who were alive at the time of DHS survey, we conduct supplementary fixed-effects analyses of monthly birth rates at the state level and individual probabilities of infant mortality. These serve to investigate for potential selection bias in our estimates of the effect of extreme heat and humidity on child height; if exposure during the period of interest is also reducing the number of children that are born or increasing rates of infant mortality, then the coefficients plotted in Fig. 2 would underestimate the true costs of prenatal exposure to one or both heat variables for child health.

Figures S7 and S8 display the results of the birth rate analysis, where each “trimester” refers to a 3-month period during the 12 months preceding the month of observation. Particularly under the relatively weaker distributional assumption of a negative binomial model, this analysis suggests that birth rates are higher than expected during the period 0 to 3 months following exposure to T_max_ > 35°C and are lower than expected 6 to 12 months after exposure to WBGT_max_ > 29°C. The former finding is consistent with prior evidence that dehydration associated with hot and dry conditions can induce labor early (*32*, *36*, *37*), so may be capturing increased rates of preterm birth for mothers and babies who were close to term when the T_max_ > 35°C exposure occurred. This shift, while important for future health outcomes, would not confer selection into our HAZ sample unless mortality occurs after birth and before the time of DHS survey. On the other hand, the WBGT_max_ effects we observe in trimesters 0 and 1 are suggestive of decreased conception rates and/or increased rates of early-stage pregnancy terminations under hot-humid conditions. This finding does imply the presence of selection bias attributable to WBGT_max_ > 29°C exposure and therefore suggests that our main estimates of the effect of hot-humid heat on child health—and the differential between WBGT_max_ and T_max_—are likely a lower bound of the true value. Meanwhile, we find no statistically detectable effect of any exposure on the probability of infant mortality (fig. S9), with very similar parameter estimates across T_max_ and WBGT_max_.

Second, we replace the DHS cluster fixed effects in our main model specifications with fixed effects at the household level. This strips out any time-invariant differences in observed and unobserved characteristics between households in our sample. Consequently, the remaining variation in the outcome and covariates come from differences between siblings within the same household, meaning that all singleton observations are dropped from the sample. The coefficient estimates on prenatal heat exposure from this model are very similar to those shown in Fig. 2, although they are estimated with less precision given the reduced sample size (*N* = 91,529) and high number of estimated parameters. These results are presented in tables S8 and S9 and fig. S13.

Third, we replicate all HAZ models using the probability of stunting as the outcome (HAZ < −2) to highlight the effects of heat exposure among those in the left tail of the height-for-age distribution (tables S10 to S12 and fig. S6). We observe the same pattern of effects from these models as from our main HAZ regressions. Moreover, working with stunting as opposed to HAZ allows us to interpret these regression results within an attributable risk framework. For instance, our model suggests that, on average, a 0.06% increase in the probability (or risk) of under-five stunting is attributable to each additional day above the mean with WBGT_max_ > 29°C in the third trimester. There is no statistically detectable change in the risk of stunting attributable to an equivalent increase in exposure to T_max_ > 35°C.

Fourth, we add a control for total precipitation at the trimester level, as precipitation is correlated with both temperature and humidity. The results from this model, shown in fig. S16, remain similar to those in Fig. 2. Fifth, we investigate the importance of our primary exposure periods (trimesters 0 to 3) by adding variables for exposure to WBGT_max_ > 29°C during the period 3 to 6 months before conception (“trimester −1”) and the 3 months after birth (“trimester 4”). The new coefficients on hot-humid heat during trimesters −1 and 4 are close to zero, whereas those on trimesters 0 to 3 remain consistent with our main results (fig. S17).

Sixth, we replicate the main results on subsets of the dataset that include only two countries at a time, dropping all observations from Bangladesh (top row of fig. S14), India (middle row), and Nepal (bottom row) in turn. All coefficients from the models without Bangladesh and without Nepal are closely aligned with those in Fig. 2, although first- and second-trimester WBGT_max_ exposures are no longer statistically different from zero at the 5% level. In the model without observations from India, which make up most of the dataset, coefficients on heat exposure are more negative across the board. This is particularly true in the case of hot-humid exposures, which are statistically significant in every trimester with magnitudes approximately two to five times larger than in the pooled model.

We also run a set of supplementary analyses to explore the sensitivity of our results to the choice of heat thresholds (T_max_ > 35°C and WBGT_max_ > 29°C). In the first of these, we construct an alternative humid heat exposure measure that classifies daily WBGT_max_ into 2°C-wide bins between 20° and 32°C. For each child, we count the total number of days per trimester in which WBGT_max_ falls into each bin for each child. We then fit a version of Eq. 2 that replaces WBGT_max_ > 29°C threshold with this bins specification (fig. S4), with days in the 20° to 22°C bin as the reference category. Given the increased number of covariates and to minimize collinearity between trimesters, we run this model for third trimester exposures only. The results from this model suggest that the magnitude of the WBGT_max_-HAZ relationship behaves fairly linearly with increasing WBGT_max_ values: the more extreme the exposure, the more negative the effect on HAZ. Third trimester exposures in the 28° to 30°C bin have a negative effect on HAZ that is statistically different from zero and is estimated with high precision relative to other bins. This suggests that our main threshold specification (29°C) is a reasonable one, consistent with previous literature (*58*, *59*).

Similarly, we construct 5°C-wide bins of T_max_ between 20° and 40°C, choosing a larger range and bin size to account for the greater spread of the T_max_ distribution compared to WBGT_max_ in our sample (fig. S1). Figure S5 presents the coefficients from a corresponding regression model that takes the 20° to 25°C bin as the reference category. As before, this model considers only third trimester exposures. The results show that, like the WBGT_max_ bins, the effect of T_max_ on HAZ becomes increasingly negative with increasing values of T_max_. Again, the relationship is approximately linear, and exposures in the highest three bins are negatively associated with HAZ and statistically different from zero. The 30° to 35°C is the first of these, and its corresponding coefficient has the smallest standard error of all T_max_ bins. Consistent with previous literature (*3*, *60*–*66*), this finding reaffirms that 35°C is an appropriate threshold choice for our primary modeling specification, as it has a statistically detectable negative effect on HAZ while still being common enough within our sample for its impact to be estimated with high precision.

Second, we replicate Eq. 2 using two locally defined percentile-based thresholds each for T_max_ and WBGT_max_. In the first of these specifications, a day is categorized as “extreme” if it exceeded the 80th percentile (or 0.8 quantile) of the historical distribution (1983 to 2016) of a given variable (T_max_ or WBGT_max_) within a given DHS cluster and calendar month. In other words, the WBGT_max_ value for a given day in January 2016 is compared to all other daily WBGT_max_ values from past Januaries in the same location. We show the 80th percentile as it most closely approximates our main absolute thresholds of WBGT_max_ = 29°C and T_max_ = 35°C, which are both close to the 0.8 quantile of their relative unconditional distributions (see fig. S1). Second, we run an equivalent version of this analysis using the 95th percentile (or 0.95 quantile), which is more commonly used in the scientific literature on climate shocks and health (*67*).

The results from these models are presented side by side with the main results from Fig. 2 in fig. S10. The coefficients on prenatal heat exposure are qualitatively consistent across the three models. In line with expectation, the coefficients on the 80th percentile exposure measures tend to be closest to those from the main specification. The effects of exposure at the 95th percentile are less precisely estimated, given that they are more rare in our sample. The 95% confidence intervals on the coefficients overlap with each other across all models and trimesters, conveying that we do not find a statistically detectable difference between them. However, we observe an increase in magnitude on third trimester Tmax exposure when moving to the percentile-based metrics, resulting in a negative effect on HAZ that is statistically different from zero. On the other hand, the negative effect of WBGT_max_ > 29°C observed in the main specification is not present in the percentile-based analysis. These changes may reflect the underlying birth seasonality in our sample, which can be seen in summary table S1. In our sample, exposures to WBGT_max_ > 29°C and T_max_ > 35°C are most rare in trimester 0 and become increasingly frequent in later trimesters, with the highest mean exposure occurring in trimester 3. While our birth month fixed effects captures a measure of birth seasonality in Eq. 2, the percentile-based measures are constructed by calendar month and therefore more explicitly adjust for this difference in exposure throughout the year.

Third, we add controls for historical averages of T_max_ and WBGT_max_ to our main specification. These means are constructed at the DHS cluster level, calculated for each calendar month, and then matched to the corresponding calendar months of each trimester for a given child. The results of this model are presented in fig. S11 and closely mirror the main results from Fig. 2. Fourth, to further alleviate concerns around the sensitivity of the primary thresholds, we replace our main exposure variables with mean monthly T_max_ and WBGT_max_ for each trimester before birth. This changes the interpretation of the coefficients such that fig. S12 now illustrates the expected change in HAZ given a one-degree increase in either T_max_ or WBGT_max_ from mean levels across each trimester. Again, the results are remarkably consistent with the pattern observable in Fig. 2.

Last, we rerun our main models using higher absolute heat thresholds (T_max_ = 40°C and WBGT_max_ = 31°C; fig. S15). While the point estimates from these models appear to suggest that these hotter conditions are less dangerous than T_max_ > 35°C and WBGT_max_ > 29°C, they suffer from a lack of precision given the rarity of these extreme values across much of our study region. These estimates are also likely subject to increases in selection bias as the risk of missed conceptions, pregnancy loss, preterm birth, and stillbirth increase at the highest heat values (*28*, *36*, *68*).

## DISCUSSION

Our results indicate that exposure to extreme heat during the year before birth undermines child health, reducing height-for-age for children under the age of five in Bangladesh, India, and Nepal. Compared to temperature alone, exposure to wet-bulb globe temperature extremes is associated with much larger reductions in height attainment, indicating a reduced ability to cope with extreme heat during humid conditions. In the third trimester, a one standard deviation increase in humid heat exposure is four times more detrimental to height attainment than an equivalent increase in exposure to heat alone, decreasing HAZ by 5.1 and 1.3% relative to the sample mean, respectively. Additional analyses of hot and hot-humid extremes’ association with birth rates reveal that each additional day with WBGT_max_ > 29°C is associated with a reduction in live births 6 to 12 months later, whereas T_max_ > 35°C is associated with an increase in the birth rate 0 to 3 months after exposure (figs. S7 and S8). In line with prior literature that links heat exposure with preterm birth (*32*, *36*, *37*), the T_max_ results suggest that high temperatures are shifting births earlier, whereas hot-humid exposures may be reducing overall conception rates, increasing rates of early-stage pregnancy loss, or both. Together, these findings suggest that our main estimates are likely a lower bound of the true effect of WBGT_max_ > 29°C exposure on child growth, as well as a lower bound of the WBGT_max_-T_max_ differential.

Our point estimates are consistent with previous research on climate-induced stunting. Whereas we find that each additional day in the third trimester with WBGT_max_ > 29°C decreases HAZ by 0.003 units (table S5), two recent studies find a 0.003-unit decrease in HAZ associated with both (i) each day of delayed monsoon onset in Indonesia and (ii) each day with extreme rain in South Asia during the prenatal period (*48*, *69*). The effects we uncover are also notable in magnitude. Combined with new climate projections, our coefficient estimates on WBGT_max_ imply that 3 to 3.7 million additional children would have been stunted in our three study countries had they been exposed to levels of heat and humidity expected by 2050 under a high-emission climate change scenario. When we define future heat exposure using T_max_, we undercount this effect by approximately 2.7 to 3.3 million children.

Our results also corroborate previous scholars’ assertations that the timing of exposure plays a critical role in determining the long-term impacts of climate shocks on child health (*70*). The adverse effects of heat exposure during trimester 3 documented above align with existing epidemiological evidence that heat stress and dehydration toward the end of gestation can induce labor prematurely, thereby increasing rates of preterm birth, low birth weights, and associated health risk for mothers and babies (*28*, *37*). In addition to providing more evidence for these mechanisms, the persistent HAZ effects that we observe imply that catch-up growth after birth is incomplete at the population level. We also find consistent and suggestive evidence that exposure to hot-humid extremes during the 3 months before conception undermines health. This added vulnerability during the preconception period suggests that maternal health status at the outset of pregnancy can influence children’s outcomes at birth and beyond. Previous research has linked extreme heat during this period with decreases in both conception rates (*68*) and birth weights (*71*). This result is also in alignment with a recent large-scale study that finds that the well-being of reproductive-age women in low- and middle-income countries is sensitive to temperature shocks across a wide range of outcomes, including nutritional health and fertility behavior (*8*). Although more research is needed to illuminate the exact mechanisms linking extreme heat before conception with height attainment after birth, the physiological consequences of extreme heat exposure during the weeks leading up to and following conception may set the most socially vulnerable children on a path toward slower growth.

In addition to magnifying the impact of heat exposure, considering humidity may provide important insights into the timing and location of heat risks. Temperature-only risk assessments may miss humid coastlines and river valleys, which are often home to dense human populations. Meanwhile, humid heat extremes tend to peak during the onset of the rainy season in monsoonal climates (*72*), thus coinciding with a particularly labor-intensive time for farmers. Together, these dynamics have implications for early warning and risk reduction efforts.

There are several limitations to our data and specification choices that could influence the analysis and findings presented here. First, the DHS variables for children’s age, month and year of birth, and length of residence in their current household rely on respondents’ recall and therefore may be subject to measurement error based on flawed memory and approximations. This potential measurement error may then affect our identification of heat exposure, which is based on exact month of birth and location of each mother within her cluster of residence during the year before giving birth. Measurement error in the variable for child’s age is also likely to affect the accuracy of HAZ, which is derived directly from age. See (*73*, *74*) for discussions on age heaping in the DHS. We are also unable to define exact trimester of exposure given that the DHS lacks complete and reliable information on day of birth and length of gestation, meaning that we necessarily take on some measurement error at the submonthly scale in our variables for the total number of hot days per trimester.

In addition, our results may be sensitive to the specification of heat thresholds and the combination of data from multiple countries, although we find the results to be consistent across six alternative heat specifications and the fixed effects in our models account for the vast majority of cross-country differences. Frequency and intensity of exposure to extreme heat and humidity are highly variable across South Asia (Fig. 1), meaning that days above our heat thresholds (T_max_ > 35°C and WBGT_max_ > 29°C) are more common in some communities than others, influencing the precision of our estimates in cooler and drier regions. While we estimate versions of the analysis with locally defined relative heat thresholds to address this, future research should take this variability into account and better address the difference in intensity and should consider the effect of consecutive versus isolated exposure to extreme heat and humidity. Moreover, we lack sufficient data on within-community differences in environmental exposures, such as air and water pollution, that may interact with heat exposure or health (*75*, *76*). Although household measures like access to an improved toilet may serve as reasonable proxies for water quality in particular (*55*), it is not possible to identify all exposures below the DHS cluster level. Point estimates from a version of the analysis with household-level fixed effects are reassuringly consistent with our main results but suffer from a lack of precision due to reduced sample size (fig. S13). Last, these data alone lack the specificity and power needed to identify or eliminate the precise mechanisms underlying the relationship between heat exposure and HAZ that we observe here.

Together, our results suggest that the long-run health consequences of extreme heat and humidity during the prenatal period represent a vastly underappreciated cost of climate change. Exposure to hot-humid extremes continues to accelerate in these regions, threatening to undermine ongoing efforts to improve child health and economic outcomes. Jobs, farms, cities, and people tend to be located along densely populated tropical river valleys and coastlines, where it is frequently very humid and likely to see increases in both heat and humidity in the coming decades. South Asian humid heat extremes, therefore, tend to follow population distributions (*50*), amplifying risk (fig. S3). Our findings point to an opportunity to protect child and maternal health from climate extremes by enhancing warning systems and medical support for pregnant people during periods of extreme heat and humidity.

## MATERIALS AND METHODS

### Heat data

We extract daily records of maximum temperature (T_max_) and maximum wet-bulb globe temperature (WBGT_max_) for each DHS survey location using the Climate Hazards Center InfraRed Temperature with Stations data (CHIRTS), a high-resolution gridded temperature product created by the Climate Hazards Center at the University of California, Santa Barbara. Specifically developed to perform well in poorly monitored regions of the Global South where weather station observations are few and far between, CHIRTS is the most robust temperature product available to date. CHIRTS improves upon previous datasets by combining satellite imagery with reanalysis data and in situ station observations to produce accurate, fine-scale (0.05° resolution) temperature estimates in otherwise data-poor regions (*29*, *30*). This product is particularly crucial in enabling our use of WBGT_max_ as a key explanatory variable. In addition to ambient air temperature, WBGT_max_ contains information about relative humidity, wind speed, and sunlight intensity, all of which influence the body’s ability to dissipate heat (*77*). In CHIRTS WBGT_max_, these influences are parameterized as a function of heat index (HI) [Eq. 1; (*77*)], which is itself calculated using CHIRTS daily T_max_ estimates and humidity estimates at the hour of local T_max_ (*56*) from the European Centre for Medium-Range Weather Forecasts Reanalysis v5 (ERA5). While the method laid out in (*78*) is now considered to be the gold standard for estimating wet-bulb globe temperature (*79*), the necessary data inputs do not exist at sufficiently high quality and with high spatial resolutions for our study region, where weather station data are only sparsely available. Instead, the CHIRTS WBGT_max_ data follow an approach (*77*) that has been shown to closely approximate shaded WBGT with fixed air speeds (0.5 m s^−1^) and is estimated as follows

$${\text{WBGT}}_{\text{max}}\left(C\right)=-0.0034*{\text{HI}}_{\text{max}}^{2}\left(F\right)+0.96*{\text{HI}}_{\text{max}}\left(F\right)-34$$ (1)

Further descriptions of the methodology for both variables and detailed validation studies can be found in (*30*, *56*).

### Defining exposure

We define extreme heat days using one biologically relevant threshold for each variable: 35°C for T_max_ and 29°C for WBGT_max_. Some existing studies use relative thresholds (usually based on percentiles of local temperature distributions or deviations from a historical mean) to account for differences in exposure and acclimatization across space (*48*, *80*). However, this strategy uses a definition of extreme heat that is not consistent across space or time of year and would include values of T_max_ and WBGT_max_ from cooler settings that—while locally or seasonally anomalous—are not likely to act on child health through the same physiological mechanisms as high heat and humidity exposure, such as extreme dehydration or heat-induced changes to placental growth (*32*, *37*, *81*). As a result, relative thresholds are most frequently used in assessing the impact of daily mean temperatures, while studies of maximum temperature tend to use absolute thresholds (*67*). Our main threshold choice follows this established convention, and we instead adjust for unequal levels of exposure and adaptation across space using cluster-level fixed effects (see the “Estimation strategy” section). In the Supplementary Materials, we show additional analyses that use relative thresholds at the 80th and 95th percentiles of the local historical distribution for T_max_ and WBGT_max_ (fig. S10) as well as explicit controls for historical T_max_ and WBGT_max_ averages at the cluster level (fig. S11).

Ambient air temperatures exceeding 35°C have been consistently shown to increase the risk of heat-induced morbidity and mortality (*3*, *60*–*66*), and wet-bulb globe temperatures above 29°C are classified as hazardous for unacclimatized people at low metabolic rates (125 to 235 W) by the US Occupational Safety and Health Administration (*58*) and the International Standards Organization (*59*). Furthermore, days with T_max_ > 35°C and WBGT_max_ > 29°C occur with nearly equal frequency (21 and 20%, respectively) in our sample, making them comparable in terms of exposure and acclimatization within the population (table S1). See figs. S4 and S5 for supplementary analyses that use a bins approach to shed additional light on the heat-growth relationship across both heat distributions.

We use information on month, year, and location of birth from the DHS to define prenatal heat exposure for each child in our sample. For each day in the CHIRTS observational period (1983 to 2016), we extract a spatial mean of all pixels that lie within a 10-km buffer around each DHS survey location. This buffer accounts for the random displacement of surveyed locations performed by the DHS to protect respondents’ privacy. Although scholars have shown that alternative geospatial approaches are sometimes better suited to accounting for such displacement in population-environment research (*82*), this buffer technique remains robust when dealing with temperature variables. We then use each child’s month and year of birth to count the number of days with T_max_ > 35°C and WBGT_max_ > 29°C during each trimester in the year before birth. In addition to trimesters 1 to 3, during which gestation takes place, we also observe heat exposure during trimester 0, which spans the 3 months preceding conception. Aside from the importance of proper maternal nutrition and health leading up to pregnancy (*70*), extreme heat during this period has been shown to reduce conceptions (*68*, *83*) and decrease the birth weights of babies born 9 to 12 months later (*27*), suggesting that these preconception months are important determinants of health trajectories for mothers and babies throughout pregnancy and beyond. Because the exact day of birth is rarely recorded and subject to recall error, we mark the start and end of each trimester using the 15th day of a given month. Although consistent across observations, this strategy means that a fraction of hot days is necessarily misassigned for any child not born on the 15th of the month. Without information on length of gestation, we also assume that each pregnancy reached a full 9 months. Last, to protect our identification of individual-level heat exposure, we exclude any respondents who moved residences at any point during the child’s life or prenatal year, as well as those lacking data on migration history altogether (*N* = 120,608).

Figure S1 depicts the distribution of daily T_max_ and WBGT_max_ for the period from 1993, when the prenatal periods for our first DHS observations begin, to the end of the CHIRTS record in 2016. The orange lines denote our final biologically relevant heat thresholds for each variable. While both distributions are left-skewed, this feature is particularly pronounced for WBGT_max_ (see fig. S1). The mode of both distributions falls around 30°C, although fig. S1B also shows a heaping of WBGT_max_ values around 22°C. For WBGT, this means that the most typical humid heat values are already potentially dangerous. This feature may reflect a seasonal shift in WBGT_max_ or else regional differences in heat and humidity across the subcontinent. In our study locations, 72.1% of days from 1993 to 2016 did not exceed either heat threshold, 12.5% exceeded both, 7.4% were extreme by T_max_ standards only, and 8.1% were extreme according only to WBGT_max_. See Fig. 1 for an understanding of how each of these four categories are distributed geographically across the region. The 15.5% of days that exceed one threshold but not the other reflect the complex construction of wet-bulb globe temperature. See figs. S18 and S19 for replications of fig. S1 that are disaggregated by country.

### Child health data

We leverage data on child growth trajectories, demographic characteristics, and community locations from the DHS. The DHS collect detailed, representative data on anthropometrics and demographics in countries that often lack adequate local and national health data. We access the DHS child questionnaires in a user-friendly format from IPUMS (*57*). Our sample includes 0- to 5-year-old children from all IPUMS-DHS surveys in Bangladesh, India, and Nepal that contain both child anthropometric records (i.e., height and weight, measured at the time of survey) and geographic identifiers at the DHS’ smallest spatial unit. These spatial units are referred to as “clusters” and are approximately the size of a single rural village or urban city block. In total, our final dataset contains 29,357 clusters with 198,710 observations from the following DHS rounds: Bangladesh 1999 to 2000 (*N* = 4260), Bangladesh 2004 (*N* = 4552), Bangladesh 2007 (*N* = 3951), India 2015 to 2016 (*N* = 174,668), Nepal 2001 (*N* = 5230), Nepal 2006 (*N* = 4229), and Nepal 2016 (*N* = 1820). Note that while DHS round-specific sampling weights help to balance unequal sample sizes between rounds, most of our observations are from India 2015 to 2016, an El Niño year with exceptionally warm Indian air temperatures (*51*).

Our outcome measure for child height is a continuous *z*-score of height-for-age ratio, which is the basis for defining stunting (HAZ < −2) (*84*). This anthropometric measurement is observed at the time of survey only. We include stunting as an additional outcome in the Supplementary Materials (see tables S10 to S12 and fig. S6). Figure S2 presents the distribution of HAZ in our sample, with markers for the thresholds of stunting and severe stunting as well as the sample median. The HAZ for each child is calculated relative to the median height among a globally representative population of children of the same age and sex (*84*) and ranges from −6 to 6 standard deviations. Notably, the sample median falls close to HAZ = −2, meaning that nearly 50% of all 0 to 5 year olds in our sample were stunted at the time of survey. In addition to HAZ, we use a number of key demographic variables related to socioeconomic status, child development, and health. See the “Estimation strategy” section for a full list of covariates and table S1 for summary statistics.

### Estimation strategy

We use two main models of child height. First, we regress height attainment on trimester-level exposure to either T_max_ > 35°C or WBGT_max_ > 29°C, along with our full suite of demographic controls and cluster, month, and state by survey-year fixed effects (Eq. 2). We run this model twice—once for T_max_ and once for WBGT_max_ extremes—and include covariates for exposure in all trimesters (0 to 3) each time. See fig. S17 for the results of an alternative model that includes exposure during the 3-month period before trimester 0 as well as the 3 months after birth. Our first regression is estimated as follows

$$Y_{\mathrm{ij}}=\beta_{0}+\sum_{\tau =0}^{\tau =3}\beta_{1_{\tau }}\;D_{h\tau \mathrm{ij}}+\beta_{2}X_{\mathrm{ij}}+\mu_{j}+\gamma_{\mathrm{ts}}+\in_{\mathrm{ij}}$$ (2)

where *Y_ij_* is the HAZ score for child *i* in DHS cluster *j*. As a supplement, we also run Eq. 2 as a linear probability model with stunting as the outcome (see tables S10 to S12 and fig. S6). The β_1_ captures the effect of a marginal increase in the number of days (*D*_*h*τ*ij*_) that the heat metric *D* exceeded threshold *h* (corresponding to T_max_ > 35°C or WBGT_max_ > 29°C) during trimester τ for child *i* in cluster *j* on our prediction of the conditional mean of HAZ, holding other covariates constant. For ease of interoperability, we standardize *D* as a *z*-score in our primary model (Fig. 2). In this case, β_1_ can be interpreted as the expected change in HAZ given a one standard deviation increase in *D*. See the Supplementary Materials for alternative versions of Eq. 2 that use different definitions of prenatal heat exposure and add controls for trimester-level precipitation and local historical exposure. The *X_ij_* term is a comprehensive set of controls at the child, maternal, and household levels. These include the child’s sex, twin status, birth order, and birth location (health clinic or other); mother’s educational attainment, parity, religion, and marital status; and an indicator variable for whether the household has access to an improved toilet (defined using DHS classifications). A set of fixed effects for child’s age in months, birth month, mother’s age in years, and month of DHS survey is also captured in *X_ij_* to account for nonlinearity in height-for-age across age groups and seasonal variation in nutritional status. Note that these fixed effects make no assumption on functional form, allowing them to vary more flexibly than a polynomial (e.g., quadratic) specification. We include also cluster fixed effects (μ*_j_*), which control for all latent and time-invariant community-level characteristics that influence children’s heights. See tables S8 and S9 and fig. S13 for a version of the analysis that replaces these cluster fixed effects with household-level fixed effects. Last, we include state-by-survey-year fixed effects (γ*_ts_*) to flexibly capture macro-level trends in child health and nutrition over time. With these robust fixed effects, the remaining variation comes from within-cluster differences in prenatal heat exposure among individuals, based on the varying ages of children in our sample. Specifically, we compare children with similar sociodemographic characteristics who were born in the same cluster and calendar month but different years within the 5-year period captured retrospectively by a given DHS survey. Having thus controlled for spatial, seasonal, and socioeconomic factors, we assume that the remaining variation in heat exposure and HAZ is random between children in the same community. We further include DHS sampling weights to account for the clustered sampling design and unequal sample sizes across countries and survey years, and we cluster standard errors at the DHS cluster level.

Our second model closely mirrors Eq. 2, but now includes covariates for trimester-level exposure to both T_max_ > 35°C (θ_τ*ij*_) and WBGT_max_ > 29°C (ω_τ*ij*_) so as to create a more direct comparison of the relative health effects of each heat type (Eq. 3).

$$Y_{\mathrm{ij}}=\beta_{0}+\sum_{\tau =0}^{\tau =3}\beta_{1_{\tau }}\;\theta_{\tau \mathrm{ij}}+\sum_{\tau =0}^{\tau =3}\beta_{2_{\tau }}\;\omega_{\tau \mathrm{ij}}+\beta_{3}X_{\mathrm{ij}}+\mu_{j}+\gamma_{\mathrm{ts}}+\in_{\mathrm{ij}}$$ (3)

Now, β_1_ represents the change in the conditional mean of the outcome given a marginal increase in the number of days where T_max_ exceeded 35°C during trimester τ for child *i* living in cluster *j* after holding hot-humid heat exposure constant, in addition to the other covariates. Likewise, β_2_ now reports the marginal effect of trimester-level exposure to days with WBGT_max_ > 29°C, unconfounded by T_max_. The excluded category therefore encompasses all days that do not surpass either heat threshold. The outcome variable, controls, and fixed-effect terms remain unchanged from Eq. 2.

Last, to investigate the presence of selection bias in our models of child height, we construct two supplementary analyses to test the effects of prenatal heat exposure on birth rates and the probability of infant mortality. Although we focus primarily on the effects of extreme heat on HAZ and stunting because of existing evidence linking them to adverse outcomes for well-being in adulthood (*44*, *47*), the epidemiological literature suggests that extreme heat during the year preceding birth may also lead to higher rates of early- and late-stage miscarriage, preterm birth, and reductions in fertility related to biological or behavioral mechanisms (*32*, *35*–*37*, *68*). Where these outcomes of extreme heat exposure exist, they may be systematically removing the children who are most vulnerable to heat from our sample, whether through increased pregnancy terminations, mortality after birth, or reductions in fertility among the most vulnerable mothers. Such selection bias would imply that our estimates of the true prenatal heat effect on child health are biased downward.

To address these concerns, we first run a state-level regression that takes the crude birth rate per month as its outcome. If selection bias is present and driven through changes to conception or pregnancy terminations, we would expect to see a decrease in births associated with a recent exposure to extreme heat and/or humidity. We aggregate our child-level dataset to the state by DHS wave level (to account for the unequal number of DHS surveys between countries) and count the number of births recorded in each state for each month in our dataset. The resulting panel of births per state contains 209,560 births, including the 198,710 children who survived until the age they were surveyed by the DHS and 10,850 children who died before survey. We then estimate a log-rate regression model of the following form

$$Y_{\mathrm{st}}=\beta_{0}+\sum_{\tau =0}^{\tau =3}\beta_{1_{\tau }}\;D_{h\tau \mathrm{st}}+\beta_{2}\text{ln}\left(P_{\mathrm{st}}\right)+\mu_{s}+\gamma_{t}+\in_{\mathrm{st}}$$ (4)

where *Y_st_* is the total number of births in state *s* during month-year *t*. As before, β_1_ represents the effect of a marginal increase in the number of days (*D*_*h*τ*st*_) that the heat metric *D* exceeded threshold *h* during trimester τ on our prediction of total births in state *s* and month-year *t*, holding other covariates constant. Now, each trimester τ can be thought of as a 3-month window during the year preceding the month of observation, such that trimester 0 represents the period 9 to 12 months ago, trimester 1 represents 6 to 9 months ago, and so on. We also include the natural logarithm of the estimated total population of women *P* living in state *s* during month-year *t*, which lends the interpretation of our coefficients as the estimated change in the crude birth rate at the state level. Our estimates of total state population come from version 4 of the Center for International Earth Science Information Network’s Gridded Population of the World dataset (*85*). These data are derived from global census inputs and made available in 5-year intervals for 2000 to 2020 as a raster with a 0.5° spatial resolution. We use zonal statistics in R to sum the estimated population living in each state using state-level shapefiles that were harmonized across survey waves by IPUMS (*57*). Next, we pair each state-month-year in our birth dataset with the most proximate estimate of total state-level population (2000, 2005, 2010, or 2015). Last, we estimate the total female population *P* using annual statistics on total population sex ratio at the country level from the United Nations Population Division (*86*). Equation 4 also includes state by DHS wave fixed effects (μ*_s_*) and month-year fixed effects (γ*_t_*). We run this model under Poisson and negative binomial distributional assumptions and present the results from both in the Supplementary Materials (figs. S7 and S8).

In addition to selection before birth, our main results may be biased downward if prenatal heat exposure has a positive effect on infant mortality, which is defined as death within the first year after birth. To that end, we also estimate a linear probability model that takes the same form as Eq. 2 but with infant mortality as the outcome. We run this individual-level model on a sample of 209,860 children, which includes the 198,710 children who survived and were present in the HAZ sample and 10,850 children who died between birth and the time of DHS survey. Of these, 9726 died within the first year of life. The results from our supplementary models of birth rates and infant mortality can be found in the Supplementary Materials (figs. S7 to S9).

## References

[1] IPCC, “AR6 synthesis report: Climate change 2023” (Tech. Rep., 2023); https://ipcc.ch/report/ar6/syr/.

[2] S. Hajat, B. G. Armstrong, N. Gouveia, P. Wilkinson, Mortality displacement of heat-related deaths: A comparison of Delhi, São Paulo, and London. Epidemiology 16, 613–620 (2005). 16135936 10.1097/01.ede.0000164559.41092.2a

[3] O. Deschenes, Temperature, human health, and adaptation: A review of the empirical literature. Energy Econ. 46, 606–619 (2014).

[4] T. Guo, Y. Wang, H. Zhang, Y. Zhang, J. Zhao, Y. Wang, X. Xie, L. Wang, Q. Zhang, D. Liu, Y. He, Y. Yang, J. Xu, Z. Peng, X. Ma, The association between ambient temperature and the risk of preterm birth in China. Sci. Total Environ. 613-614, 439–446 (2018). 28918275 10.1016/j.scitotenv.2017.09.104

[5] B. A. Fletcher, S. Lin, E. F. Fitzgerald, S.-A. Hwang, Association of summer temperatures with hospital admissions for renal diseases in New York State: A case-crossover study. Am. J. Epidemiol. 175, 907–916 (2012). 22455834 10.1093/aje/kwr417

[6] F. B. Nerbass, R. Pecoits-Filho, W. F. Clark, J. M. Sontrop, C. W. McIntyre, L. Moist, Occupational heat stress and kidney Health: From farms to factories. Kidney Int. Rep. 2, 998–1008 (2017). 29270511 10.1016/j.ekir.2017.08.012 PMC5733743

[7] D. G. Smith, “What extreme heat does to your body,” *The New York Times*, 9 August 2023; https://nytimes.com/interactive/2023/08/10/well/live/heat-body-dehydration-health.html.

[8] C. Gray, B. Thiede, Temperature anomalies undermine the health of reproductive-age women in low- and middle-income countries. Proc. Natl. Acad. Sci. U.S.A. 121, e2311567121 (2024). 38442166 10.1073/pnas.2311567121 PMC10945799

[9] C. Mora, B. Dousset, I. R. Caldwell, F. E. Powell, R. C. Geronimo, C. R. Bielecki, C. W. W. Counsell, B. S. Dietrich, E. T. Johnston, L. V. Louis, M. P. Lucas, M. M. McKenzie, A. G. Shea, H. Tseng, T. W. Giambelluca, L. R. Leon, E. Hawkins, C. Trauernicht, Global risk of deadly heat. Nat. Clim. Change 7, 501–506 (2017).

[10] A. Hsu, G. Sheriff, T. Chakraborty, D. Manya, Disproportionate exposure to urban heat island intensity across major US cities. Nat. Commun. 12, 2721 (2021). 34035248 10.1038/s41467-021-22799-5 PMC8149665

[11] L. Cushing, R. Morello-Frosch, A. Hubbard, Extreme heat and its association with social disparities in the risk of spontaneous preterm birth. Paediatr. Perinat. Epidemiol. 36, 13–22 (2022). 34951022 10.1111/ppe.12834

[12] J. Vanos, G. Guzman-Echavarria, J. W. Baldwin, C. Bongers, K. L. Ebi, O. Jay, A physiological approach for assessing human survivability and liveability to heat in a changing climate. Nat. Commun. 14, 7653 (2023). 38030628 10.1038/s41467-023-43121-5 PMC10687011

[13] D. P. Lakhoo, N. Brink, L. Radebe, M. H. Craig, M. D. Pham, M. M. Haghighi, A. Wise, I. Solarin, S. Luchters, G. Maimela, M. F. Chersich, Heat-Health Study Group, HIGH Horizons Study Group, A systematic review and meta-analysis of heat exposure impacts on maternal, fetal and neonatal health. Nat. Med. 31, 684–694 (2025). 39500369 10.1038/s41591-024-03395-8 PMC11835737

[14] K. Parsons, *Human Thermal Environments: The Effects of Hot, Moderate, and Cold Environments on Human Health, Comfort and Performance, Second Edition* (CRC Press, ed. 2, 2002).

[15] G. Havenith, D. Fiala, Thermal indices and thermophysiological modeling for heat stress. Compr. Physiol. 6, 255–302 (2016). 10.1002/cphy.c140051 26756633

[16] D. J. Vecellio, S. T. Wolf, R. M. Cottle, W. L. Kenney, Evaluating the 35°C wet-bulb temperature adaptability threshold for young, healthy subjects (PSU HEAT Project). J. Appl. Physiol. 132, 340–345 (2022). 34913738 10.1152/japplphysiol.00738.2021 PMC8799385

[17] N. T. Vargas, C. L. Chapman, W. Ji, B. D. Johnson, R. Gathercole, Z. J. Schlader, Increased skin wetness independently augments cool-seeking behaviour during passive heat stress. J. Physiol. 598, 2775–2790 (2020). 32347543 10.1113/JP279537

[18] R. G. Steadman, The assessment of sultriness. Part I: A temperature-humidity index based on human physiology and clothing science. J. Appl. Meteorol. Climatol. 18, 861–873 (1979).

[19] K. Parsons, Heat stress standard ISO 7243 and its global application. Ind. Health 44, 368–379 (2006). 16922180 10.2486/indhealth.44.368

[20] G. M. Budd, Wet-bulb globe temperature (WBGT)–Its history and its limitations. J. Sci. Med. Sport 11, 20–32 (2008). 17765661 10.1016/j.jsams.2007.07.003

[21] J. Schwartz, J. M. Samet, J. A. Patz, Hospital admissions for heart disease: The effects of temperature and humidity. Epidemiology 15, 755–761 (2004). 15475726 10.1097/01.ede.0000134875.15919.0f

[22] A. Barnett, S. Tong, A. C. Clements, What measure of temperature is the best predictor of mortality? Environ. Res. 110, 604–611 (2010). 20519131 10.1016/j.envres.2010.05.006

[23] G. B. Anderson, M. L. Bell, R. D. Peng, Methods to calculate the heat index as an exposure metric in environmental health research. Environ. Health Perspect. 121, 1111–1119 (2013). 23934704 10.1289/ehp.1206273 PMC3801457

[24] B. Armstrong, F. Sera, A. M. Vicedo-Cabrera, R. Abrutzky, D. O. Åström, M. L. Bell, B.-Y. Chen, M. de Sousa Zanotti Stagliorio Coelho, P. M. Correa, T. N. Dang, M. H. Diaz, D. Van Dung, B. Forsberg, P. Goodman, Y.-L. L. Guo, Y. Guo, M. Hashizume, Y. Honda, E. Indermitte, C. Íñiguez, H. Kan, H. Kim, J. Kyselý, E. Lavigne, P. Michelozzi, H. Orru, N. V. Ortega, M. Pascal, M. S. Ragettli, P. H. N. Saldiva, J. Schwartz, M. Scortichini, X. Seposo, A. Tobias, S. Tong, A. Urban, C. De la Cruz Valencia, A. Zanobetti, A. Zeka, A. Gasparrini, The role of humidity in associations of high temperature with mortality: A multicountry, multicity study. Environ. Health Perspect. 127, 097007 (2019). 31553655 10.1289/EHP5430 PMC6792461

[25] J. W. Baldwin, T. Benmarhnia, K. L. Ebi, O. Jay, N. J. Lutsko, J. K. Vanos, Humidity’s role in heat-related health outcomes: A heated debate. Environ. Health Perspect. 131, 055001 (2023). 37255302 10.1289/EHP11807 PMC10231239

[26] E.-S. Im, J. S. Pal, E. A. B. Eltahir, Deadly heat waves projected in the densely populated agricultural regions of South Asia. Sci. Adv. 3, e1603322 (2017). 28782036 10.1126/sciadv.1603322 PMC5540239

[27] K. Grace, F. Davenport, H. Hanson, C. Funk, S. Shukla, Linking climate change and health outcomes: Examining the relationship between temperature, precipitation and birth weight in Africa. Glob. Environ. Change 35, 125–137 (2015).

[28] F. Davenport, A. Dorélien, K. Grace, Investigating the linkages between pregnancy outcomes and climate in sub-Saharan Africa. Popul. Environ. 41, 397–421 (2020). 39391542 10.1007/s11111-020-00342-w PMC11465627

[29] C. Funk, P. Peterson, S. Peterson, S. Shukla, F. Davenport, J. Michaelsen, K. R. Knapp, M. Landsfeld, G. Husak, L. Harrison, J. Rowland, M. Budde, A. Meiburg, T. Dinku, D. Pedreros, N. Mata, A high-resolution 1983–2016 *T*_max_ climate data record based on infrared temperatures and stations by the climate hazard center. J. Clim. 32, 5639–5658 (2019).

[30] A. Verdin, C. Funk, P. Peterson, M. Landsfeld, C. Tuholske, K. Grace, Development and validation of the CHIRTS-daily quasi-global high-resolution daily temperature data set. Sci. Data 7, 303 (2020). 32929097 10.1038/s41597-020-00643-7 PMC7490712

[31] D. J. Vecellio, Q. Kong, W. L. Kenney, M. Huber, Greatly enhanced risk to humans as a consequence of empirically determined lower moist heat stress tolerance. Proc. Natl. Acad. Sci. U.S.A. 120, e2305427120 (2023). 37812703 10.1073/pnas.2305427120 PMC10589700

[32] C. M. Stan, M. Boulvain, R. Pfister, P. Hirsbrunner-Almagbaly, Hydratioen for treatment of preterm labour. Cochrane Database Syst. Rev. 2013, CD003096 (2013). 24190310 10.1002/14651858.CD003096.pub2 PMC11751767

[33] M. Carolan-Olah, D. Frankowska, High environmental temperature and preterm birth: A review of the evidence. Midwifery 30, 50–59 (2014). 23473912 10.1016/j.midw.2013.01.011

[34] Y. Zhang, C. Yu, L. Wang, Temperature exposure during pregnancy and birth outcomes: An updated systematic review of epidemiological evidence. Environ. Pollut. 225, 700–712 (2017). 28284544 10.1016/j.envpol.2017.02.066

[35] R. Basu, R. Rau, D. Pearson, B. Malig, Temperature and term low birth weight in California. Am. J. Epidemiol. 187, 2306–2314 (2018). 29901701 10.1093/aje/kwy116

[36] A. Ward, J. Clark, J. McLeod, R. Woodul, H. Moser, C. Konrad, The impact of heat exposure on reduced gestational age in pregnant women in North Carolina, 2011-2015. Int. J. Biometeorol. 63, 1611–1620 (2019). 31367892 10.1007/s00484-019-01773-3

[37] H. Randell, C. Gray, K. Grace, Stunted from the start: Early life weather conditions and child undernutrition in Ethiopia. Soc. Sci. Med. 261, 113234 (2020). 32823214 10.1016/j.socscimed.2020.113234 PMC7716344

[38] J. Sexton, C. Andrews, S. Carruthers, S. Kumar, V. Flenady, S. Lieske, Systematic review of ambient temperature exposure during pregnancy and stillbirth: Methods and evidence. Environ. Res. 197, 111037 (2021). 33781772 10.1016/j.envres.2021.111037

[39] S. Petrou, T. Sach, L. Davidson, The long-term costs of preterm birth and low birth weight: Results of a systematic review. Child Care Health Dev. 27, 97–115 (2001). 11251610 10.1046/j.1365-2214.2001.00203.x

[40] D. Moster, R. T. Lie, T. Markestad, Long-term medical and social consequences of preterm birth. N. Engl. J. Med. 359, 262–273 (2008). 18635431 10.1056/NEJMoa0706475

[41] G. K. Swamy, T. Østbye, R. Skjærven, Association of preterm birth with long-term survival, reproduction, and next-generation preterm birth. JAMA 299, 1429–1436 (2008). 18364485 10.1001/jama.299.12.1429

[42] A. M. Ahmed, S. M. Grandi, E. Pullenayegum, S. D. McDonald, M. Beltempo, S. S. Premji, J. D. Pole, F. Bacchini, P. S. Shah, P. Pechlivanoglou, Short-term and long-term mortality risk after preterm birth. JAMA Netw. Open 7, e2445871 (2024). 39565625 10.1001/jamanetworkopen.2024.45871 PMC11579792

[43] P. Rayco-Solon, A. J. Fulford, A. M. Prentice, Maternal preconceptional weight and gestational length. Am. J. Obstet. Gynecol. 192, 1133–1136 (2005). 15846192 10.1016/j.ajog.2004.10.636

[44] D. Almond, J. Currie, Killing me softly: The fetal origins hypothesis. J. Econ. Perspect. 25, 153–172 (2011). 25152565 10.1257/jep.25.3.153 PMC4140221

[45] U. Ramakrishnan, F. Grant, T. Goldenberg, A. Zongrone, R. Martorell, Effect of women’s nutrition before and during early pregnancy on maternal and infant outcomes: A systematic review. Paediatr. Perinat. Epidemiol. 26, 285–301 (2012). 22742616 10.1111/j.1365-3016.2012.01281.x

[46] J. Nobles, A. Hamoudi, Detecting the effects of early-life exposures: Why fecundity matters. Popul. Res. Policy Rev. 38, 783–809 (2019). 33408430 10.1007/s11113-019-09562-x PMC7785096

[47] H. Alderman, J. Hoddinott, B. Kinsey, Long term consequences of early childhood malnutrition. Oxf. Econ. Pap. 58, 450–474 (2006).

[48] K. McMahon, C. Gray, Climate change, social vulnerability and child nutrition in South Asia. Glob. Environ. Change 71, 102414 (2021). 34898861 10.1016/j.gloenvcha.2021.102414 PMC8653856

[49] R. K. Pachauri, M. R. Allen, V. R. Barros, J. Broome, W. Cramer, R. Christ, J. A. Church, L. Clarke, Q. Dahe, P. Dasgupta, N. K. Dubash, O. Edenhofer, I. Elgizouli, C. B. Field, P. Forster, P. Friedlingstein, J. Fuglestvedt, L. Gomez-Echeverri, S. Hallegatte, G. Hegerl, M. Howden, K. Jiang, B. Jimenez Cisneroz, V. Kattsov, H. Lee, K. J. Mach, J. Marotzke, M. D. Mastrandrea, L. Meyer, J. Minx, Y. Mulugetta, K. O’Brien, M. Oppenheimer, J. J. Pereira, R. Pichs-Madruga, G.-K. Plattner, H.-O. Pörtner, S. B. Power, B. Preston, N. H. Ravindranath, A. Reisinger, K. Riahi, M. Rusticucci, R. Scholes, K. Seyboth, Y. Sokona, R. Stavins, T. F. Stocker, P. Tschakert, D. van Vuuren, J.-P. van Ypserle, *Climate Change 2014: Synthesis Report. Contribution of Working Groups I, II and III to the Fifth Assessment Report of the Intergovernmental Panel on Climate Change* (IPCC) (2014), https://epic.awi.de/id/eprint/37530/, pages: 151 Publication Title: EPIC3Geneva, Switzerland, IPCC, 151 p., pp. 151, ISBN: 978-92-9169-143-2.

[50] C. Tuholske, K. Caylor, C. Funk, A. Verdin, S. Sweeney, K. Grace, P. Peterson, T. Evans, Global urban population exposure to extreme heat. Proc. Natl. Acad. Sci. U.S.A. 118, e2024792118 (2021). 34607944 10.1073/pnas.2024792118 PMC8521713

[51] C. C. Funk, *Drought, Flood, Fire: How Climate Change Contributes to Catastrophes* (Cambridge Univ. Press, 2021).

[52] T. K. R. Matthews, R. L. Wilby, C. Murphy, Communicating the deadly consequences of global warming for human heat stress. Proc. Natl. Acad. Sci. U.S.A. 114, 3861–3866 (2017). 28348220 10.1073/pnas.1617526114 PMC5393218

[53] UNICEF, “Undernourished and overlooked: A global nutrition crisis in adolescent girls and women,” *UNICEF Child Nutrition Report Series, 2022* (2023).

[54] G. Shively, C. Sununtnasuk, M. Brown, Environmental variability and child growth in Nepal. Health Place 35, 37–51 (2015). 26183566 10.1016/j.healthplace.2015.06.008

[55] D. Spears, Exposure to open defecation can account for the Indian enigma of child height. J. Dev. Econ. 146, 102277 (2020). 32904726 10.1016/j.jdeveco.2018.08.003 PMC7457703

[56] E. Williams, C. Funk, P. Peterson, C. Tuholske, High resolution climate change observations and projections for the evaluation of heat-related extremes. Sci. Data 11, 261 (2024). 38429277 10.1038/s41597-024-03074-w PMC11422495

[57] E. H. Boyle, M. King, M. Sobek, IPUMS -Demographic and Health Surveys: Version 9 [dataset] (2022), 10.18128/D080.V9.

[58] OSHA, OSHA Technical Manual, Section III, Chapter 4. Heat Stress. (2017), https://osha.gov/dts/osta/otm/otm-iii/otm-iii-4.html.

[59] ISO, *Ergonomics of the Thermal Environment—Assessment of Heat Stress Using the WBGT (Wet Bulb Globe Temperature) Index* (Int. Org. Standard, 2017).

[60] W. Huang, H. Kan, S. Kovats, The impact of the 2003 heat wave on mortality in Shanghai, China. Sci. Total Environ. 408, 2418–2420 (2010). 20219235 10.1016/j.scitotenv.2010.02.009

[61] S. Tong, C. Ren, N. Becker, Excess deaths during the 2004 heatwave in Brisbane, Australia. Int. J. Biometeorol. 54, 393–400 (2010). 20049484 10.1007/s00484-009-0290-8

[62] M. Nitschke, G. R. Tucker, A. L. Hansen, S. Williams, Y. Zhang, P. Bi, Impact of two recent extreme heat episodes on morbidity and mortality in Adelaide, South Australia: A case-series analysis. Environ. Health 10, 42 (2011). 21592410 10.1186/1476-069X-10-42 PMC3116460

[63] S. Williams, M. Nitschke, P. Weinstein, D. L. Pisaniello, K. A. Parton, P. Bi, The impact of summer temperatures and heatwaves on mortality and morbidity in Perth, Australia 1994–2008. Environ. Int. 40, 33–38 (2012). 22280925 10.1016/j.envint.2011.11.011

[64] Y. Jun, H. Z. Liu, C. Q. Ou, G. Z. Lin, D. Yan, Z. Qin, J. C. Shen, P. Y. Chen, Impact of heat wave in 2005 on mortality in Guangzhou, China. Biomed. Environ. Sci. 26, 647–654 (2013). 23981550 10.3967/0895-3988.2013.08.003

[65] X. Sun, Q. Sun, X. Zhou, X. Li, M. Yang, A. Yu, F. Geng, Heat wave impact on mortality in Pudong New Area, China in 2013. Sci. Total Environ. 493, 789–794 (2014). 25000574 10.1016/j.scitotenv.2014.06.042

[66] A. Barreca, K. Clay, O. Deschenes, M. Greenstone, J. S. Shapiro, Adapting to climate change: The remarkable decline in the US temperature-mortality relationship over the twentieth century. J. Pol. Econ. 124, 105–159 (2016).

[67] Z. Xu, G. FitzGerald, Y. Guo, B. Jalaludin, S. Tong, Impact of heatwave on mortality under different heatwave definitions: A systematic review and meta-analysis. Environ. Int. 89, 193–203 (2016). 26878285 10.1016/j.envint.2016.02.007

[68] A. Barreca, O. Deschenes, M. Guldi, Maybe next month? Temperature shocks and dynamic adjustments in birth rates. Demography 55, 1269–1293 (2018). 29968058 10.1007/s13524-018-0690-7 PMC7457515

[69] B. C. Thiede, C. Gray, Climate exposures and child undernutrition: Evidence from Indonesia. Soc. Sci. Med. 265, 113298 (2020). 32932006 10.1016/j.socscimed.2020.113298 PMC7738425

[70] K. Grace, A. Verdin, A. Dorélien, F. Davenport, C. Funk, G. Husak, Exploring strategies for investigating the mechanisms linking climate and individual-level child health outcomes: An analysis of birth weight in Mali. Demography 58, 499–526 (2021). 33834220 10.1215/00703370-8977484 PMC8382135

[71] K. Grace, N. N. Nagle, Using high-resolution remotely sensed data to examine the relationship between agriculture and fertility in Mali. Prof. Geogr. 67, 641–654 (2015).

[72] C. Raymond, T. Matthews, R. M. Horton, The emergence of heat and humidity too severe for human tolerance. Sci. Adv. 6, eaaw1838 (2020). 32494693 10.1126/sciadv.aaw1838 PMC7209987

[73] M. Lyons-Amos, T. Stones, Trends in Demographic and Health Survey data quality: An analysis of age heaping over time in 34 countries in Sub Saharan Africa between 1987 and 2015. BMC. Res. Notes 10, 760 (2017). 29262857 10.1186/s13104-017-3091-x PMC5738749

[74] M. Singh, G. C. Kashyap, M. Bango, Age heaping among individuals in selected South Asian countries: Evidence from demographic and health surveys. J. Biosoc. Sci. 54, 725–734 (2022). 34099079 10.1017/S0021932021000249

[75] A. Hyder, H. J. Lee, K. Ebisu, P. Koutrakis, K. Belanger, M. L. Bell, PM_2.5_ exposure and birth outcomes: Use of satellite- and monitor-based data. Epidemiology 25, 58–67 (2014). 24240652 10.1097/EDE.0000000000000027 PMC4009503

[76] E. DeFranco, W. Moravec, F. Xu, E. Hall, M. Hossain, E. N. Haynes, L. Muglia, A. Chen, Exposure to airborne particulate matter during pregnancy is associated with preterm birth: A population-based cohort study. Environ. Health 15, 6 (2016). 26768419 10.1186/s12940-016-0094-3 PMC4714531

[77] T. E. Bernard, I. Iheanacho, Heat index and adjusted temperature as surrogates for wet bulb globe temperature to screen for occupational heat stress. J. Occup. Environ. Hyg. 12, 323–333 (2015). 25616731 10.1080/15459624.2014.989365

[78] J. C. Liljegren, R. A. Carhart, P. Lawday, S. Tschopp, R. Sharp, Modeling the wet bulb globe temperature using standard meteorological measurements. J. Occup. Environ. Hyg. 5, 645–655 (2008). 18668404 10.1080/15459620802310770

[79] Q. Kong, M. Huber, Explicit calculations of wet-bulb globe temperature compared with approximations and why it matters for labor productivity. Earth’s Future 10, e2021EF002334 (2022).

[80] Q. Yin, J. Wang, Z. Ren, J. Li, Y. Guo, Mapping the increased minimum mortality temperatures in the context of global climate change. Nat. Commun. 10, 4640 (2019). 31604931 10.1038/s41467-019-12663-y PMC6789034

[81] W. Cowell, N. Ard, T. Herrera, E. A. Medley, L. Trasande, Ambient temperature, heat stress and fetal growth: A review of placenta-mediated mechanisms. Mol. Cell. Endocrinol. 576, 112000 (2023). 37460007 10.1016/j.mce.2023.112000

[82] K. Grace, N. N. Nagle, C. R. Burgert-Brucker, S. Rutzick, D. C. Van Riper, T. Dontamsetti, T. Croft, Integrating environmental context into DHS analysis while protecting participant confidentiality: A new remote sensing method. Popul. Dev. Rev. 45, 197–218 (2019). 30983647 10.1111/padr.12222 PMC6446718

[83] D. A. Lam, J. A. Miron, The effects of temperature on human fertility. Demography 33, 291–305 (1996). 8875063

[84] WHO, *WHO Child Growth Standards* (World Health Organization, 2006).

[85] C. for International Earth Science Information Network-CIESIN-Columbia University, Gridded population of the world, version 4 (GPWv4): Population density, revision 11 (2018).

[86] D. o. E. United Nations, P. D. Social Affairs, World Population Prospects: The Revision (2024).

