# Supplementary material for: Does humidity matter? Prenatal heat and child health in South Asia
Source: Sci Adv. 2025 Dec 19;11(51):eadx3010. doi: 10.1126/sciadv.adx3010 (PMC12716390; doi:10.1126/sciadv.adx3010)
Supplement: Supplementary file 1 — Figs. S1 to S20 Tables S1 to S12 [file sciadv.adx3010_sm.pdf]

Science Advances
AAAS

Supplementary Materials for

Does humidity matter? Prenatal heat and child health in South Asia

Kathryn McMahon *et al.*

Corresponding author: Kathryn McMahon, kathryn.mcmahon@geog.ucsb.edu

Sci. Adv. **11**, eadx3010 (2025)
DOI: 10.1126/sciadv.adx3010

This PDF file includes:

Figs. S1 to S20
Tables S1 to S12

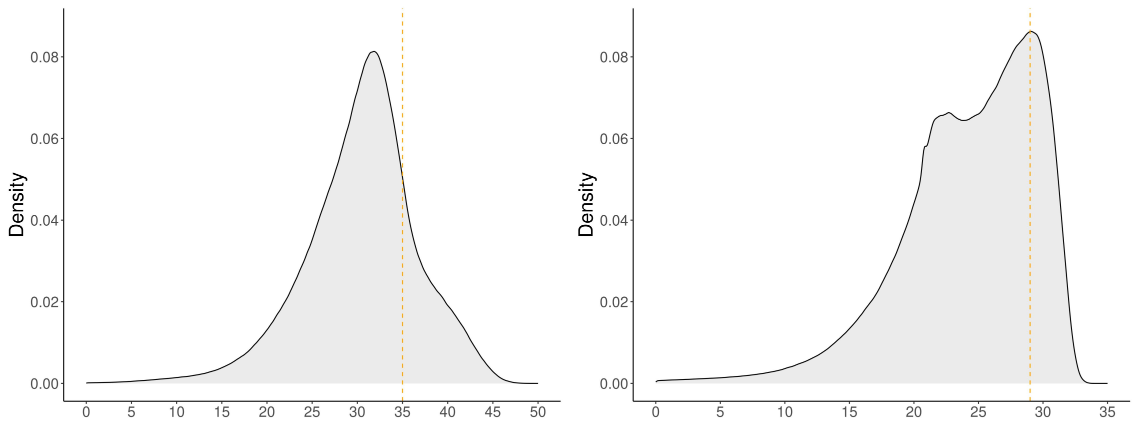

(A) Tmax

(B) WBGTmax

Figure S1: **Density curves of daily heat in all DHS clusters (1993-2016).** Panel (**A**) shows the distribution of Tmax values, and Panel (**B**) depicts the WBGTmax distribution. Orange dotted lines mark our biologically-relevant thresholds (Tmax=35°C, WBGTmax=29°C).

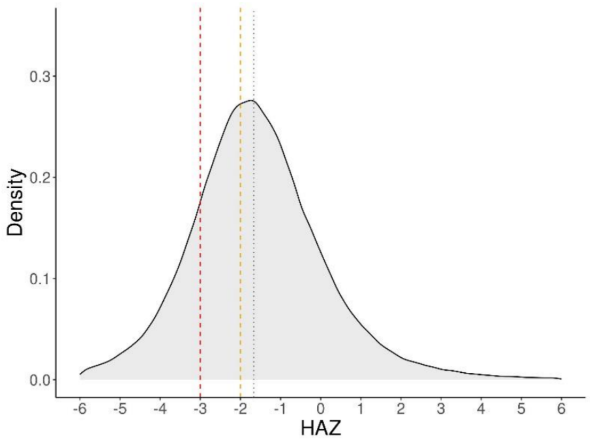

Figure S2: **Density curve of HAZ in DHS sample.** Black dotted line marks the sample median. Orange and red dashed lines mark the WHO thresholds for stunting and severe stunting, respectively.

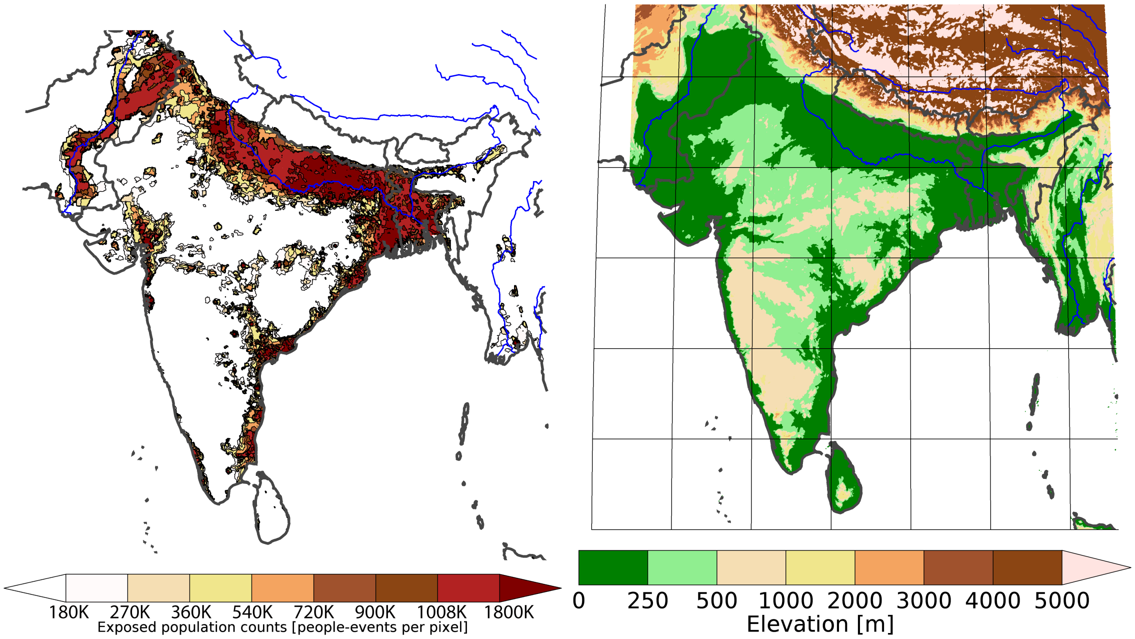

(A) (B)

Figure S3: **Geophysical context of South Asia.** Panel (**A**) uses gridded population data from WorldPop to show total exposure (in person-days per pixel) to wet-bulb globe temperatures exceeding 30°C in India, Bangladesh, Nepal, and Pakistan from 1983-2016. Panel (**B**) depicts elevation in meters and major rivers across the region.

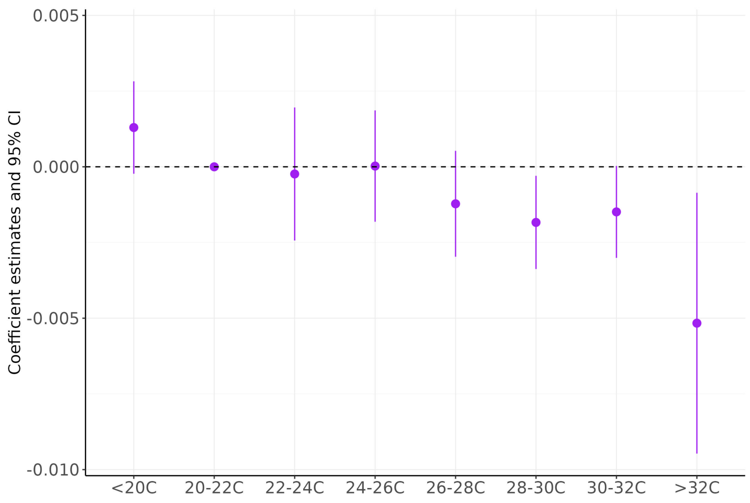

Figure S4: **HAZ results for trimester 3 using binned WBGTmax specification.** Coefficients and 95% confidence intervals for the effect of an additional day in a given WBGTmax bin during trimester 3 on HAZ. Days with WBGTmax between 20°C and 22°C are the reference category. Controls for child's sex, twin status, birth order, birth location, child's age in months, birth month, month of survey, mother's age in years, mother's educational attainment, parity, religion, marital status, and improved toilet access are included in the model but not shown. Fixed effects for cluster and state-by-survey-year are also included in the model but not shown.

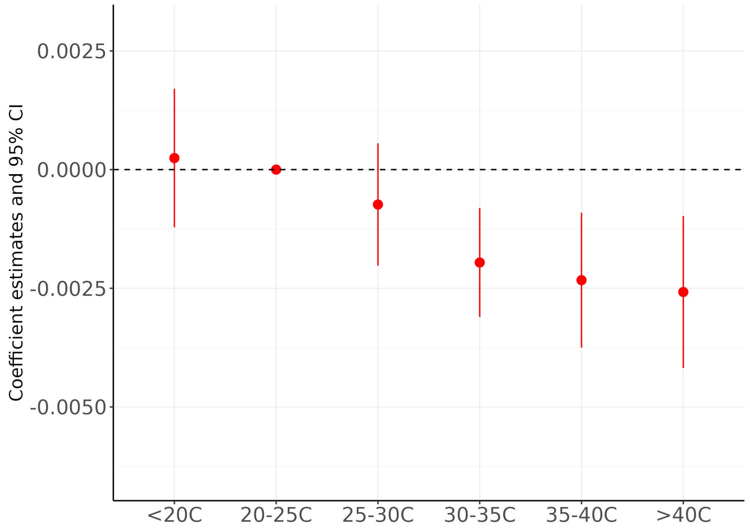

Figure S5: **HAZ results for trimester 3 using binned Tmax specification.** Coefficients and 95% confidence intervals for the effect of an additional day in a given Tmax bin during trimester 3 on HAZ. Days with Tmax between 20°C and 25°C are the reference category. Controls for child's sex, twin status, birth order, birth location, child's age in months, birth month, month of survey, mother's age in years, mother's educational attainment, parity, religion, marital status, and improved toilet access are included in the model but not shown. Fixed effects for cluster and state-by-survey-year are also included in the model but not shown.

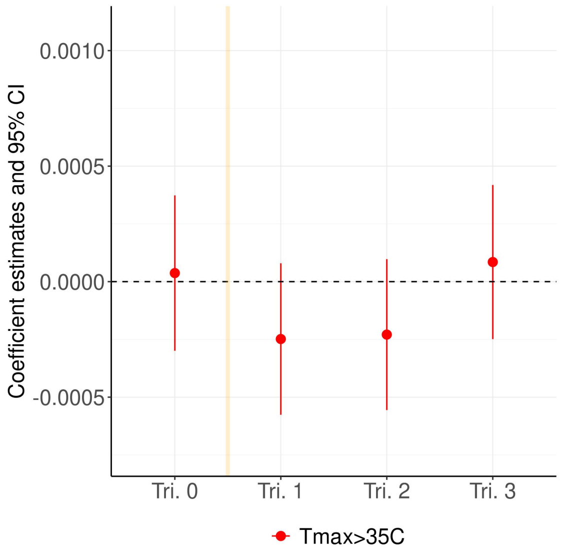

(A) Tmax

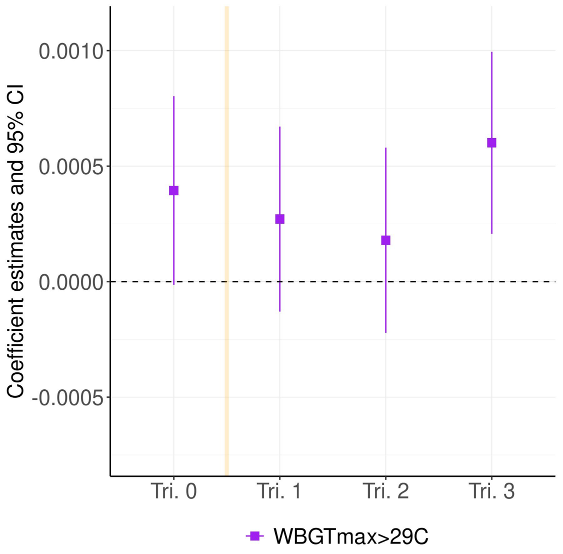

(B) WBGTmax

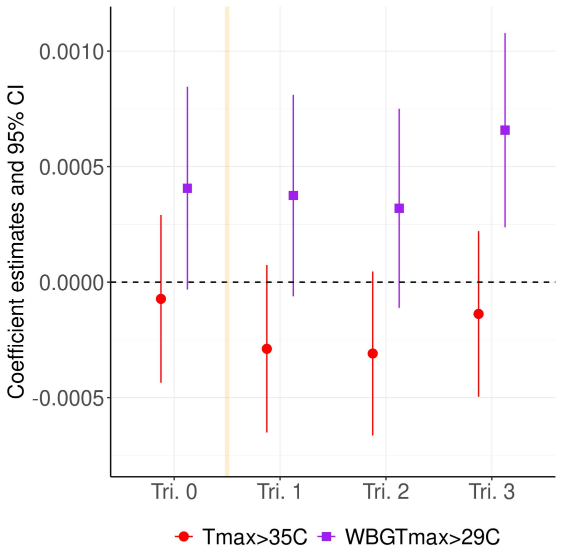

(C) Both

Figure S6: **Main effects of heat exposure on stunting.** Coefficients and 95% confidence intervals for the effect of Tmax>35°C (Panel **A**), WBGTmax>29°C (Panel **B**), and all heat (Panel **C**) on the probability of being stunted. ”Tri. 0” refers to the three-month period before conception, which is represented in each plot with a yellow vertical line. Controls for child’s sex, twin status, birth order, birth location, child’s age in months, birth month, month of survey, mother’s age in years, mother’s educational attainment, parity, religion, marital status, and improved toilet access are included in the model but not shown. Fixed effects for cluster and state-by-survey-year are also omitted (see tables S10-S12 for full regression results).

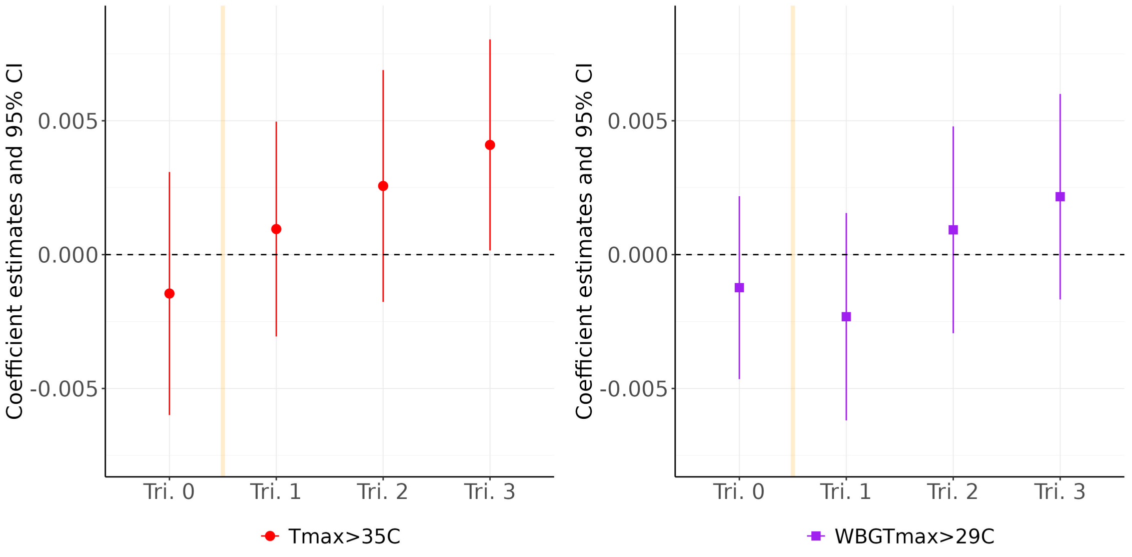

(A) Tmax (B) WBGTmax

Figure S7: **State-level effects of heat exposure on crude birth rate (Poisson model).** Coefficients and 95% confidence intervals for the estimated effect of Tmax>35°C (Panel **A**) and WBGTmax>29°C (Panel **B**) on the monthly crude birth rate at the Admin 1 (or state) level. Here, each trimester refers to a three-month period in the year preceding the month of observation. "Tri. 0" refers to the period 9-12 months before the month of observation. In each plot, the yellow vertical line denotes 9 months before the current month, representing the start of gestation for full-term babies born in the current month. Fixed effects for year, calendar month, and state-by-survey are included in the model but not shown.

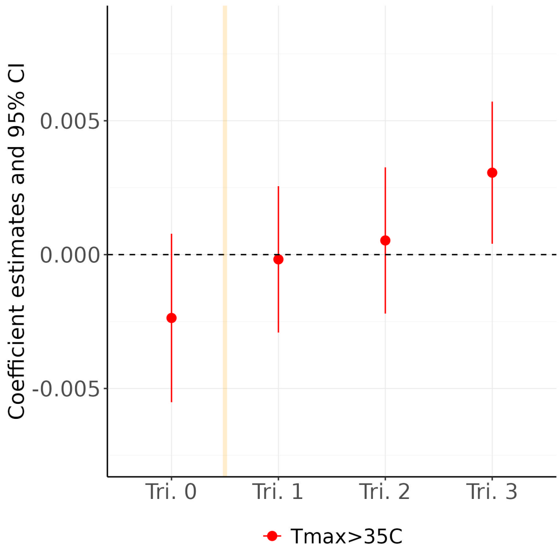

(A) Tmax

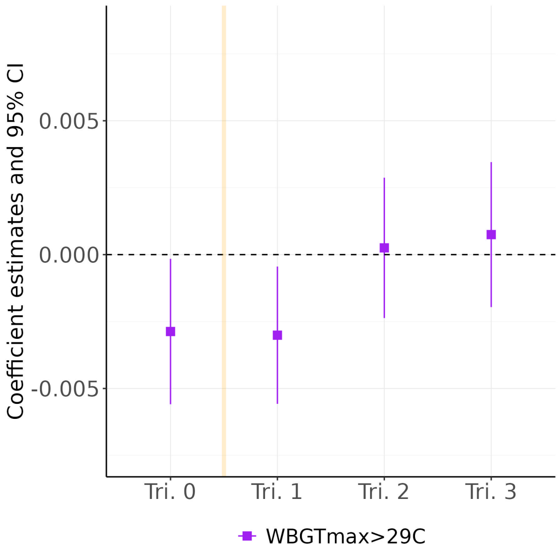

(B) WBGTmax

Figure S8: **State-level effects of heat exposure on crude birth rate (Negative binomial model).** Coefficients and 95% confidence intervals for the estimated effect of Tmax>35°C (Panel **A**) and WBGTmax>29°C (Panel **B**) on the monthly crude birth rate at the Admin 1 (or state) level. Here, each trimester refers to a three-month period in the year preceding the month of observation. ”Tri. 0” refers to the period 9-12 months before the month of observation. In each plot, the yellow vertical line denotes 9 months before the current month, representing the start of gestation for full-term babies born in the current month. Fixed effects for year, calendar month, and state-by-survey are included in the model but not shown.

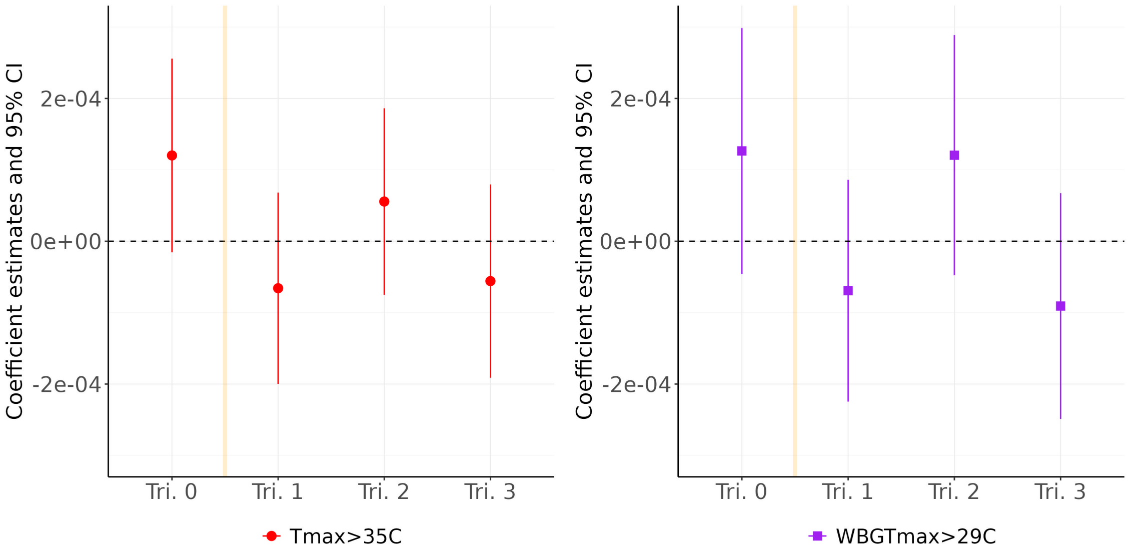

(A) Tmax (B) WBGTmax

Figure S9: **Main effects of heat exposure on infant mortality.** Coefficients and 95% confidence intervals for the effect of Tmax>35°C (Panel **A**) and WBGTmax>29°C (Panel **B**) on the probability of death within the first year after birth. "Tri. 0" refers to the three-month period before conception, which is represented in each plot with a yellow vertical line. Controls for child's sex, twin status, birth order, birth location, child's age in months (realized or counterfactual), birth month, month of survey, mother's age in years, mother's educational attainment, parity, religion, marital status, and improved toilet access are included in the model but not shown. Fixed effects for cluster and state-by-survey-year are also omitted.

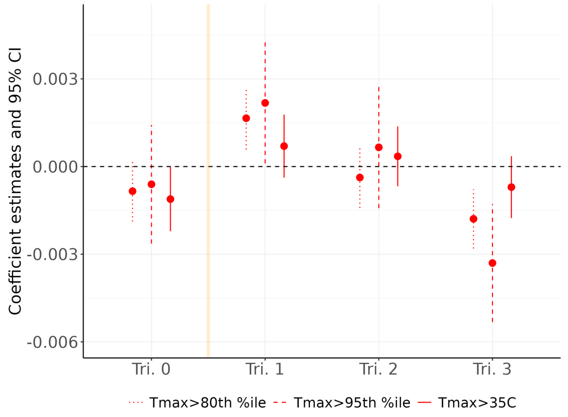

(A) Tmax

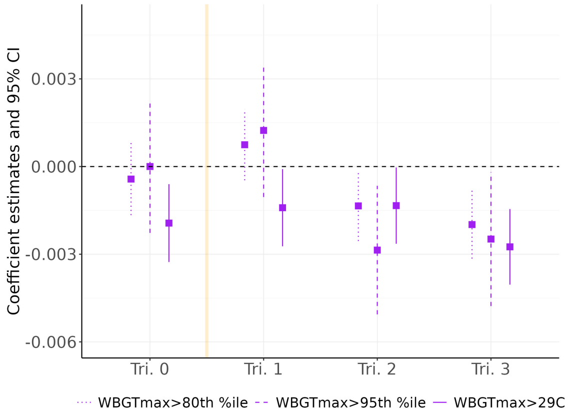

(B) WBGTmax

Figure S10: **Main effects of heat exposure on HAZ using absolute versus relative heat thresholds.** Dotted and dashed lines show the coefficients and 95% confidence intervals for the effect of one additional day with Tmax>80th percentile and Tmax>95th percentile (Panel **A**) and WBGTmax>80th percentile WBGTmax>95th percentile (Panel **B**) on HAZ, respectively. Solid lines show the same coefficients and 95% confidence intervals presented in the main specification (Figure 2). ”Tri. 0” refers to the three-month period before conception, which is represented in each plot with a yellow vertical line. Controls for child’s sex, twin status, birth order, birth location, child’s age in months, birth month, month of survey, mother’s age in years, mother’s educational attainment, parity, religion, marital status, and improved toilet access are included in the model but not shown. Fixed effects for cluster and state-by-survey-year are also omitted.

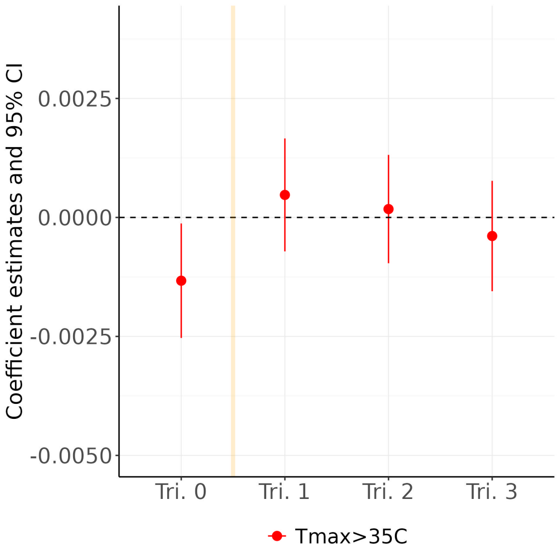

(A) Tmax

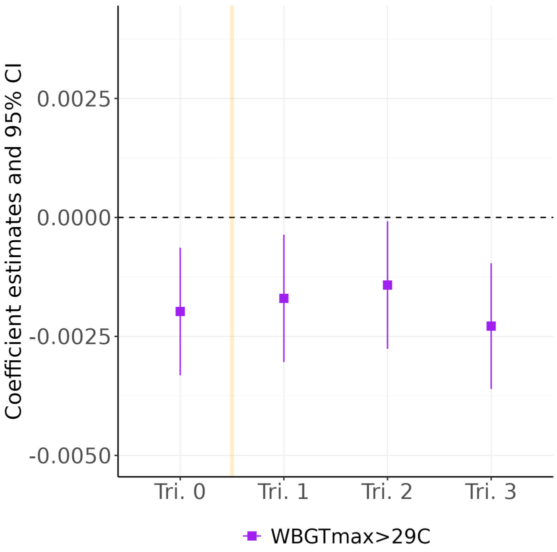

(B) WBGTmax

Figure S11: **HAZ results after controlling for historical heat exposure.** Coefficients and 95% confidence intervals for the effect of Tmax>35°C (Panel **A**) and WBGTmax>29°C (Panel **B**) on HAZ after controlling for historical means of Tmax, WBGTmax, or both variables, respectively. The means were constructed using 34 years of data (1983-2016) for each calendar month at the DHS cluster level. "Tri. 0" refers to the three-month period before conception, which is represented in each plot with a yellow vertical line. Controls for child's sex, twin status, birth order, birth location, child's age in months, birth month, month of survey, mother's age in years, mother's educational attainment, parity, religion, marital status, and improved toilet access are included in the model but not shown. Fixed effects for cluster and state-by-survey-year are also omitted.

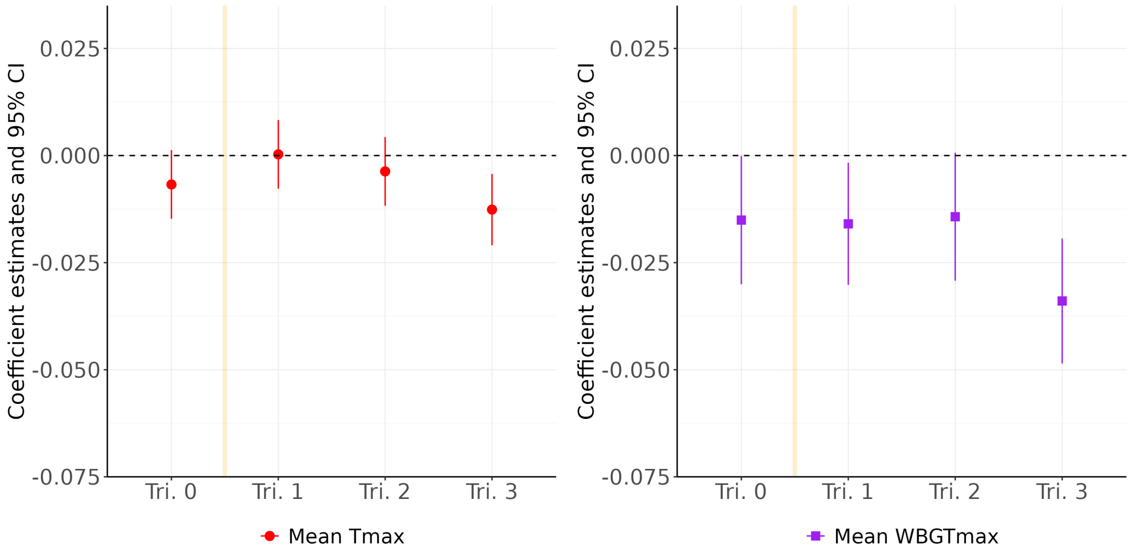

(A) Tmax (B) WBGTmax

Figure S12: **Effects of mean heat exposure on HAZ.** Coefficients and 95% confidence intervals for the effect of mean daily Tmax (Panel **A**) and mean WBGTmax (Panel **B**) in each trimester on HAZ. "Tri. 0" refers to the three-month period before conception, which is represented in each plot with a yellow vertical line. Controls for child's sex, twin status, birth order, birth location, child's age in months, birth month, month of survey, mother's age in years, mother's educational attainment, parity, religion, marital status, and improved toilet access are included in the model but not shown. Fixed effects for cluster and state-by-survey-year are also omitted.

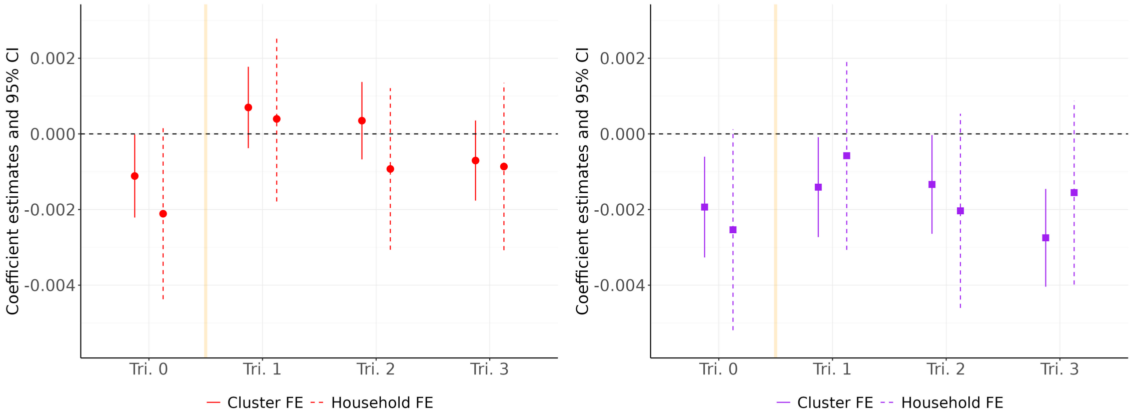

(A) Tmax (B) WBGTmax

Figure S13: **Main effects of heat exposure on HAZ with cluster versus household-level fixed effects.** Dashed lines show the coefficients and 95% confidence intervals for the effect of Tmax>35°C (Panel **A**) and WBGTmax>29°C (Panel **B**) on HAZ from a model with household fixed effects. Solid lines show the same coefficients and 95% confidence intervals presented in the main specification, which includes fixed effects for DHS cluster and not for household (Figure 2). ”Tri. 0” refers to the three-month period before conception, which is represented in each plot with a yellow vertical line. Controls for child’s sex, twin status, birth order, birth location, child’s age in months, birth month, month of survey, mother’s age in years, mother’s educational attainment, parity, religion, marital status, and improved toilet access are included in the model but not shown. (see tables S8-S9 for regression results).

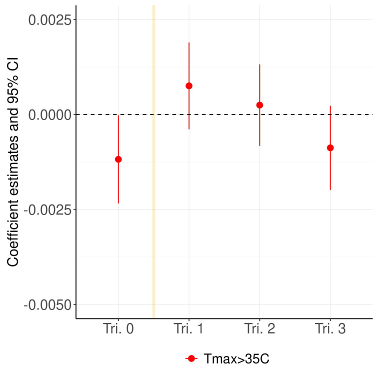

(A) Without Bangladesh

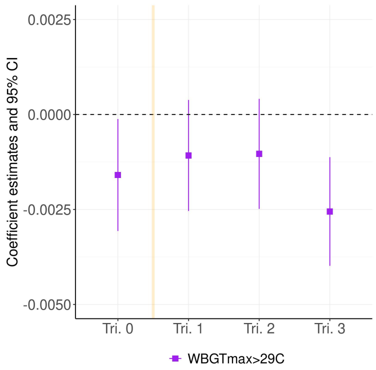

(B) Without Bangladesh

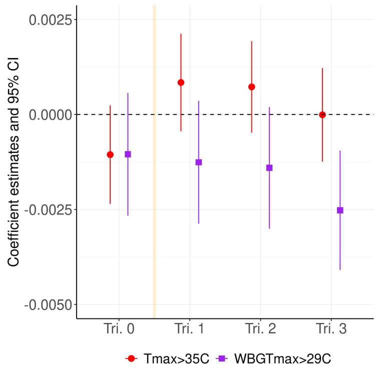

(C) Without Bangladesh

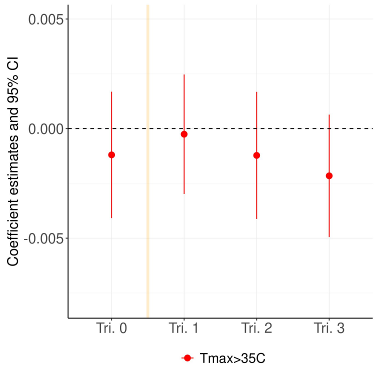

(D) Without India

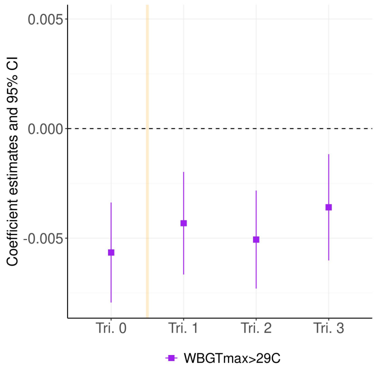

(E) Without India

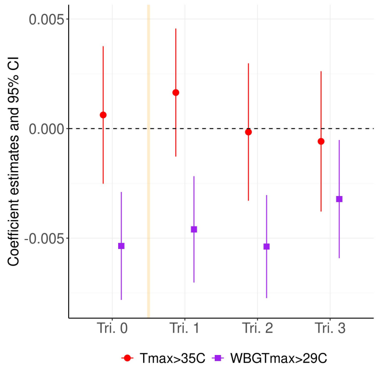

(F) Without India

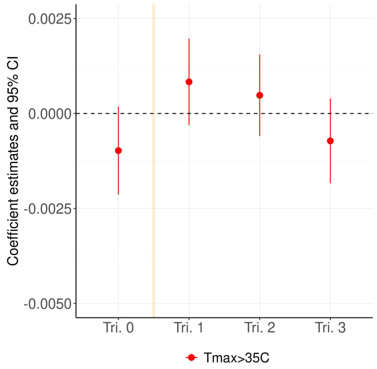

(G) Without Nepal

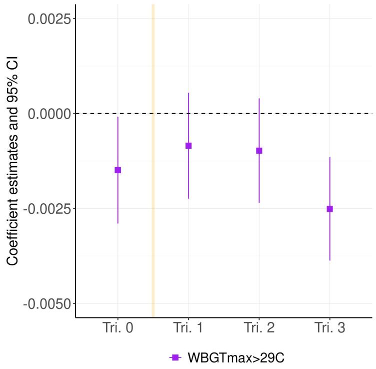

(H) Without Nepal

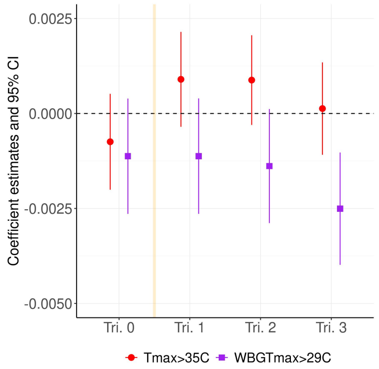

(I) Without Nepal

Figure S14: **Main effects of heat exposure on HAZ when each country is removed from the sample.** Top, middle, and bottom rows replicate the main results without observations from Bangladesh, India, and Nepal, respectively. In each row, coefficients and 95% confidence intervals for the effect of Tmax>35°C (Panels **A**, **D**, and **G**), WBGTmax>29°C (Panels **B**, **E**, and **H**), and all heat (Panels **C**, **F**, and **I**) on HAZ. "Tri. 0" refers to the three-month period before conception, which is represented in each plot with a yellow vertical line. Controls for child's sex, twin status, birth order, birth location, child's age in months, birth month, month of survey, mother's age in years, mother's educational attainment, parity, religion, marital status, and improved toilet access are included in the model but not shown. Fixed effects for cluster and state-by-survey-year are also omitted.

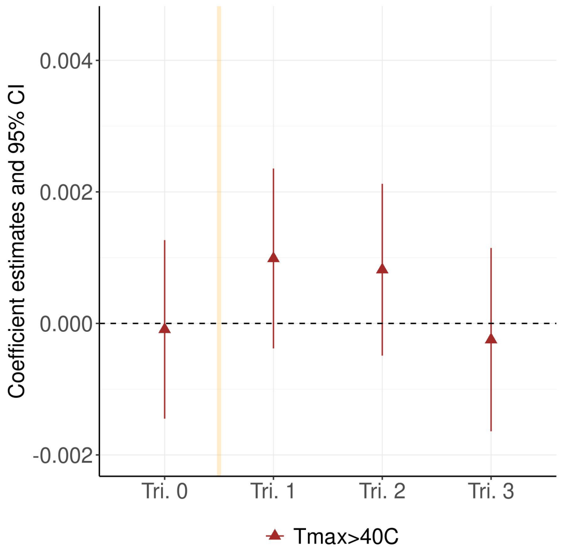

(A) Tmax

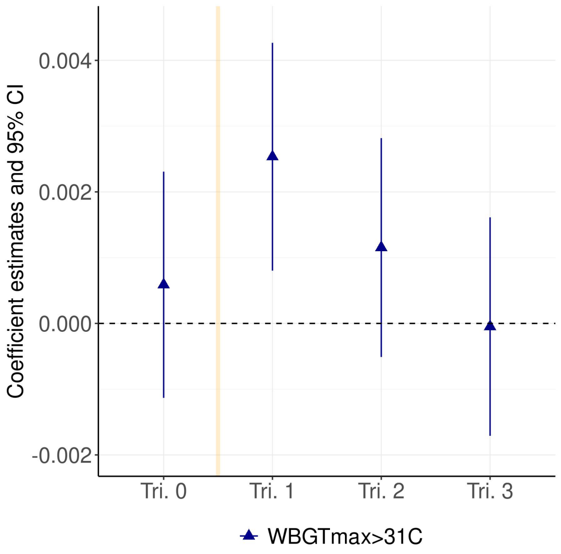

(B) WBGTmax

Figure S15: **HAZ results using alternative heat thresholds.** Coefficients and 95% confidence intervals for the effect of Tmax>40°C (Panel **A**) and WBGTmax>31°C (Panel **B**) on HAZ. "Tri. 0" refers to the three-month period before conception, which is represented in each plot with a yellow vertical line. Controls for child's sex, twin status, birth order, birth location, child's age in months, birth month, month of survey, mother's age in years, mother's educational attainment, parity, religion, marital status, and improved toilet access are included in the model but not shown. Fixed effects for cluster and state-by-survey-year are also included in the model but not shown.

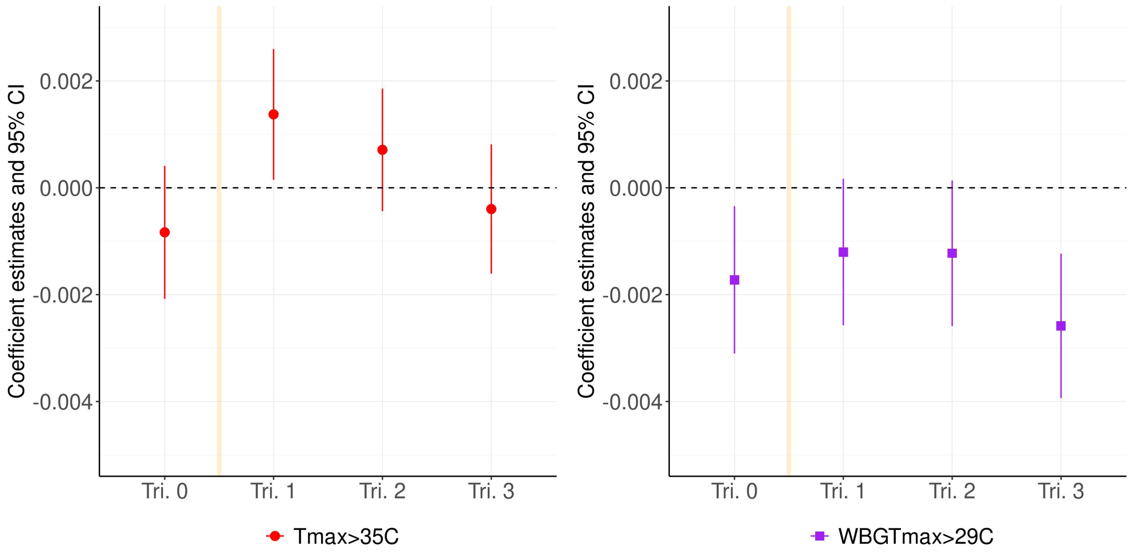

(A) Tmax

(B) WBGTmax

Figure S16: **HAZ results after controlling for trimester-level precipitation.** Coefficients and 95% confidence intervals for the effect of Tmax>35°C (Panel **A**) and WBGTmax>29°C (Panel **B**) on HAZ. ”Tri. 0” refers to the three-month period before conception, which is represented in each plot with a yellow vertical line. Controls for total precipitation per trimester, child’s sex, twin status, birth order, birth location, child’s age in months, birth month, month of survey, mother’s age in years, mother’s educational attainment, parity, religion, marital status, and improved toilet access are included in the model but not shown. Fixed effects for cluster and state-by-survey-year are also included in the model but not shown.

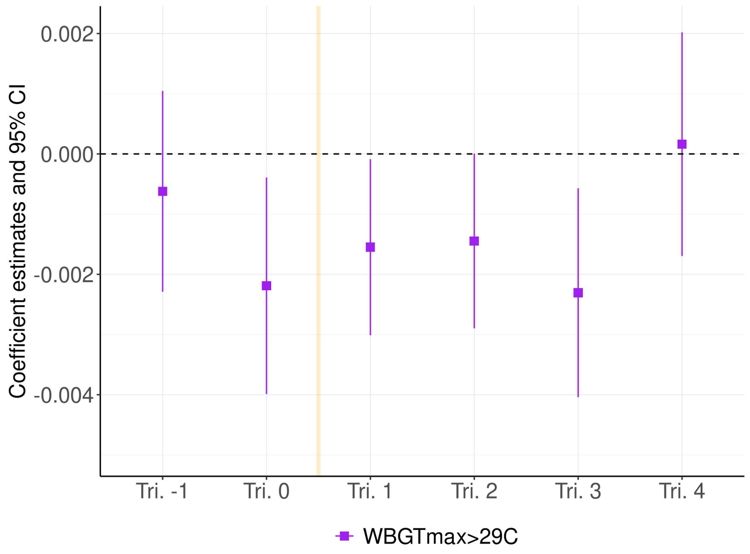

Figure S17: **HAZ results with additional exposure periods.** Coefficients and 95% confidence intervals for the effect of WBGTmax>29°C on HAZ. ”Tri. 0” refers to the three-month period before conception, which is represented in each plot with a yellow vertical line. ”Tri. -1” refers to the period 3-6 months before conception, while ”Tri. 4” refers to the first three months of life after birth. Controls for child’s sex, twin status, birth order, birth location, child’s age in months, birth month, month of survey, mother’s age in years, mother’s educational attainment, parity, religion, marital status, and improved toilet access are included in the model but not shown. Fixed effects for cluster and state-by-survey-year are also included in the model but not shown.

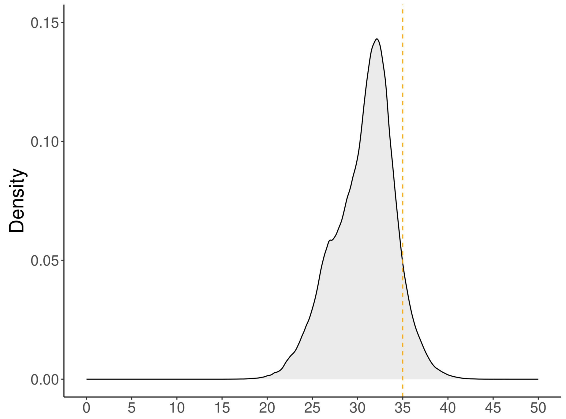

(A) Bangladesh

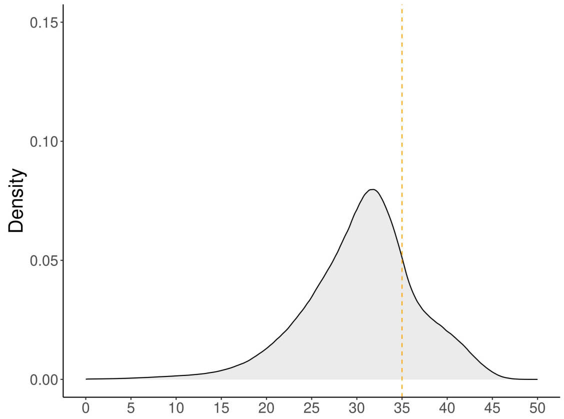

(B) India

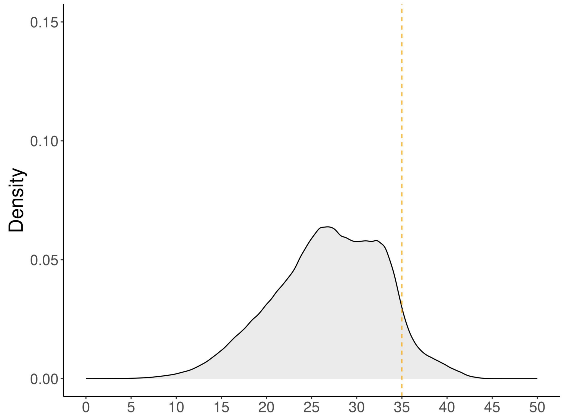

(C) Nepal

Figure S18: **Density curves of daily Tmax by country**. Distributions presented for all DHS clusters in Bangladesh (Panel **A**), India (Panel **B**), and Nepal (Panel **C**) during the period 1993-2016. Orange dotted line marks our biologically-relevant threshold (Tmax=35°C).

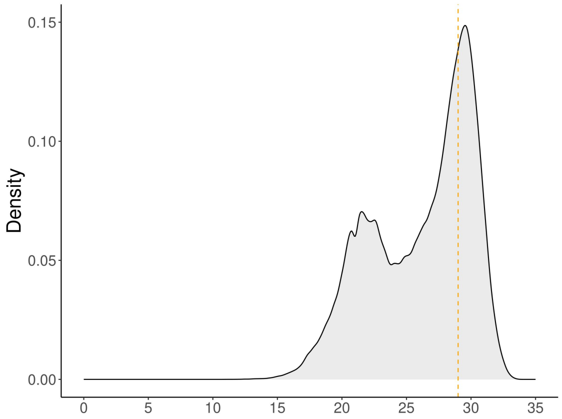

(A) Bangladesh

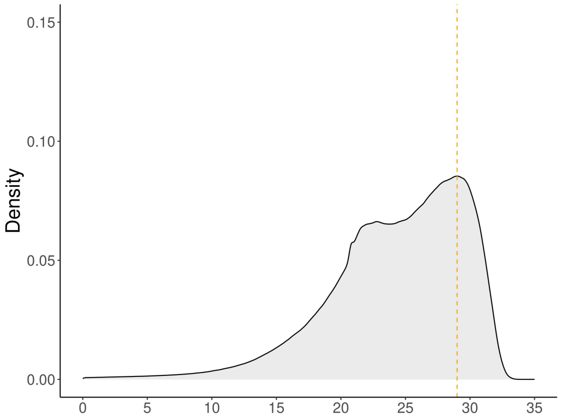

(B) India

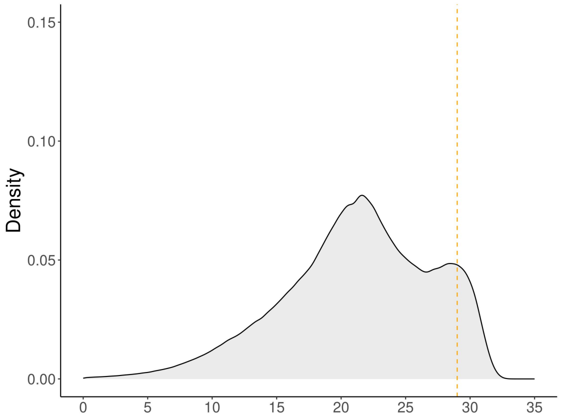

(C) Nepal

Figure S19: **Density curves of daily WBGTmax by country**. Distributions presented for all DHS clusters in Bangladesh (Panel **A**), India (Panel **B**), and Nepal (Panel **C**) during the period 1993-2016. Orange dotted line marks our biologically-relevant threshold (WBGTmax=29°C).

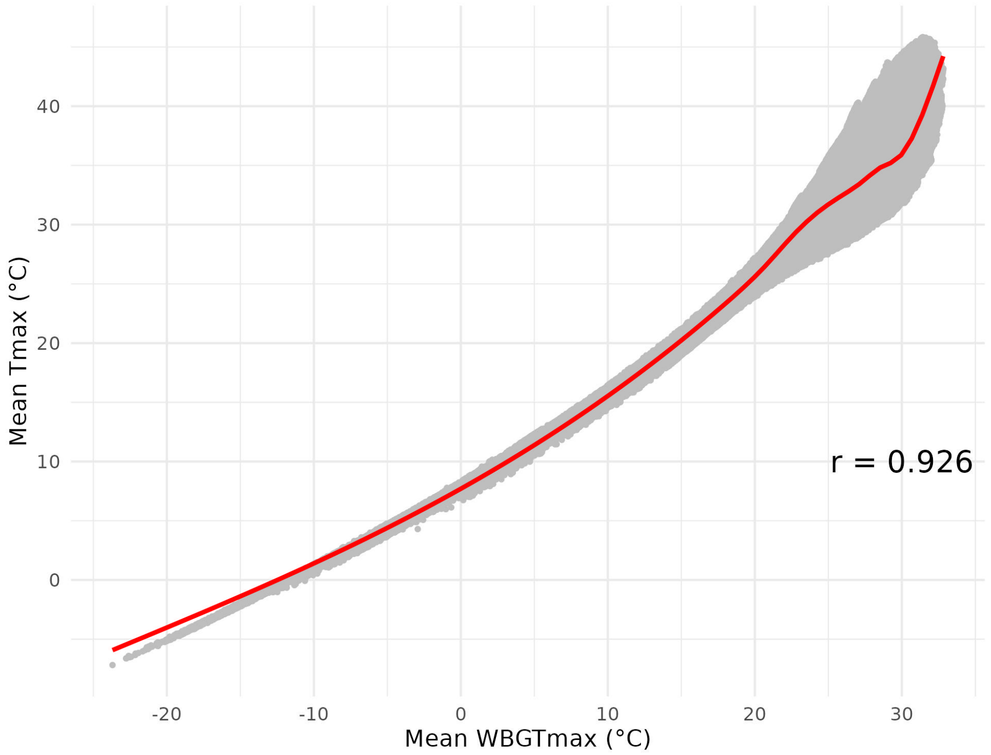

Figure S20: **Scatterplot of mean monthly WBGTmax versus Tmax at the DHS cluster level.** Units are in degrees Celsius, data are from 1993-2016. A smooth trend line is plotted in red. The Pearson correlation coefficient (r) on the data shown is reported in the bottom right-hand corner.

Table S1: **Unweighted summary statistics for explanatory variables and outcomes (N=198,710).**

	Mean	SD	Min	Median	Max
**Child Demographics**					
Height-for-age	-1.57	1.67	-6.00	-1.67	6.00
Stunted	0.41	0.49	0.00	0.00	1.00
Severely stunted	0.18	0.38	0.00	0.00	1.00
Age (months)	30.82	16.95	1.00	31.00	60.00
Birth order	2.55	1.59	1.00	2.00	16.00
Twin	0.01	0.12	0.00	0.00	1.00
Female	0.48	0.50	0.00	0.00	1.00
Birth location					
Clinic	0.66	0.47	0.00	1.00	1.00
Home	0.33	0.47	0.00	0.00	1.00
Other	0.00	0.06	0.00	0.00	1.00
**Maternal Demographics**					
Stunted	0.53	0.50	0.00	1.00	1.00
Age	27.63	5.33	13.00	27.00	49.00
Parity	2.82	1.62	1.00	2.00	16.00
Married	0.99	0.11	0.00	1.00	1.00
Educational Attainment					
None	0.36	0.48	0.00	0.00	1.00
Primary	0.16	0.37	0.00	0.00	1.00
Secondary	0.40	0.49	0.00	0.00	1.00
Higher	0.07	0.26	0.00	0.00	1.00
Religion					
Christian	0.07	0.25	0.00	0.00	1.00
Hindu	0.70	0.46	0.00	1.00	1.00

Muslim	0.20	0.40	0.00	0.00	1.00
Buddhist	0.01	0.11	0.00	0.00	1.00
Sikh	0.01	0.11	0.00	0.00	1.00
Jain	0.00	0.03	0.00	0.00	1.00
Improved toilet	0.53	0.50	0.00	1.00	1.00
Urban	0.21	0.41	0.00	0.00	1.00
**Prenatal Heat Exposure**					
Observed					
Days with Tmax>35C					
Trimester 0	17.50	25.16	0.00	3.00	92.00
Trimester 1	18.51	25.82	0.00	3.00	92.00
Trimester 2	20.70	26.78	0.00	6.00	92.00
Trimester 3	19.75	26.24	0.00	5.00	92.00
Days with WBGTmax>29C					
Trimester 0	19.24	26.21	0.00	2.00	92.00
Trimester 1	19.03	26.32	0.00	2.00	92.00
Trimester 2	21.66	27.47	0.00	5.00	92.00
Trimester 3	22.35	28.08	0.00	5.00	92.00
Projected with 2050 Warming (SSP2-4.5)					
Days with Tmax>35C					
Trimester 0	23.65	27.95	0.00	11.00	92.00
Trimester 1	24.53	28.57	0.00	11.00	92.00
Trimester 2	27.15	29.37	0.00	16.00	92.00
Trimester 3	26.25	28.98	0.00	15.00	92.00
Days with WBGTmax>29C					
Trimester 0	29.60	32.66	0.00	15.00	92.00
Trimester 1	29.08	32.73	0.00	13.00	92.00

Trimester 2	32.44	33.63	0.00	21.00	92.00
Trimester 3	33.17	34.11	0.00	22.00	92.00
Projected with 2050 Warming (SSP5-8.5)					
Days with Tmax>35C					
Trimester 0	25.56	28.51	0.00	14.00	92.00
Trimester 1	26.38	29.12	0.00	15.00	92.00
Trimester 2	29.09	29.85	0.00	19.00	92.00
Trimester 3	28.17	29.49	0.00	18.00	92.00
Days with WBGTmax>29C					
Trimester 0	32.19	33.78	0.00	20.00	92.00
Trimester 1	31.60	33.86	0.00	18.00	92.00
Trimester 2	35.09	34.64	0.00	25.00	92.00
Trimester 3	35.80	35.08	0.00	26.00	92.00

Table S2: **HAZ regression results from Tmax-only model with standard errors.** Tmax variables have been transformed as Z-scores. Controls for twin status, mother's parity, mother's marital status, religion, child's birth month, mother's age in years, survey month, and child's age in months are included in the model but not shown. See Eq. 2 for full model details.

Dependent Variable:	Height-for-age Z-score (HAZ)			
	Tmax > 35C			
Model:	(1)	(2)	(3)	(4)
Variables				
Female (child)	0.06***	0.06***	0.06***	0.06***
	(0.010)	(0.010)	(0.010)	(0.010)
Birth place (ref=Clinic)				
Home	-0.04***	-0.04***	-0.04***	-0.04***
	(0.01)	(0.01)	(0.01)	(0.01)
Other	-0.04	-0.04	-0.04	-0.04
	(0.07)	(0.07)	(0.07)	(0.07)
Mother's Education (ref=Higher)				
None	-0.49***	-0.49***	-0.50***	-0.50***
	(0.03)	(0.03)	(0.03)	(0.03)
Primary	-0.41***	-0.41***	-0.41***	-0.41***
	(0.03)	(0.03)	(0.03)	(0.03)
Secondary	-0.24***	-0.24***	-0.24***	-0.24***
	(0.03)	(0.03)	(0.03)	(0.03)
Improved Toilet	0.22***	0.22***	0.22***	0.22***
	(0.01)	(0.01)	(0.01)	(0.01)
Number of extreme days (Z-score)				
Trimester 3	-0.03**	-0.02**	-0.004	-0.02
	(0.01)	(0.01)	(0.01)	(0.01)
Trimester 2		0.02**	0.03***	0.009

	(1)	(2)	(3)	(4)
		(0.01)	(0.01)	(0.01)
Trimester 1			0.03^{***}	0.02
			(0.01)	(0.01)
Trimester 0				-0.03^{**}
				(0.01)
Fixed-effects				
DHS cluster	Yes	Yes	Yes	Yes
State-survey year	Yes	Yes	Yes	Yes
Fit statistics				
Observations	198,710	198,710	198,710	198,710
R^2	0.33919	0.33923	0.33928	0.33930
Within R^2	0.01834	0.01839	0.01846	0.01850

Clustered (DHS cluster) standard-errors in parentheses

*Signif. Codes: ***: 0.01, **: 0.05, *: 0.1*

Table S3: **HAZ regression results from non-standardized Tmax-only model with standard errors.** Controls for twin status, mother's parity, mother's marital status, religion, child's birth month, mother's age in years, survey month, and child's age in months are included in the model but not shown. See Eq. 2 for full model details.

Dependent Variable:	Height-for-age Z-score (HAZ)			
	Tmax > 35C			
Model:	(1)	(2)	(3)	(4)
Variables				
Female (child)	0.06***	0.06***	0.06***	0.06***
	(0.010)	(0.010)	(0.010)	(0.010)
Birth place (ref=Clinic)				
Home	-0.04***	-0.04***	-0.04***	-0.04***
	(0.01)	(0.01)	(0.01)	(0.01)
Other	-0.04	-0.04	-0.04	-0.04
	(0.07)	(0.07)	(0.07)	(0.07)
Mother's Education (ref=Higher)				
None	-0.49***	-0.49***	-0.50***	-0.50***
	(0.03)	(0.03)	(0.03)	(0.03)
Primary	-0.41***	-0.41***	-0.41***	-0.41***
	(0.03)	(0.03)	(0.03)	(0.03)
Secondary	-0.24***	-0.24***	-0.24***	-0.24***
	(0.03)	(0.03)	(0.03)	(0.03)
Improved Toilet	0.22***	0.22***	0.22***	0.22***
	(0.01)	(0.01)	(0.01)	(0.01)
Number of extreme days				
Trimester 3	-0.0009**	-0.0008**	-0.0001	-0.0007
	(0.0004)	(0.0004)	(0.0005)	(0.0005)
Trimester 2		0.0008**	0.001***	0.0003

		(0.0004)	(0.0004)	(0.0005)
Trimester 1			0.001***	0.0007
			(0.0005)	(0.0006)
Trimester 0				-0.001**
				(0.0006)
Fixed-effects				
DHS cluster	Yes	Yes	Yes	Yes
State-survey year	Yes	Yes	Yes	Yes
Fit statistics				
Observations	198,710	198,710	198,710	198,710
R^2	0.33919	0.33923	0.33928	0.33930
Within R^2	0.01834	0.01839	0.01846	0.01850

Clustered (DHS cluster) standard-errors in parentheses

*Signif. Codes: ***: 0.01, **: 0.05, *: 0.1*

Table S4: **HAZ regression results from WBGTmax-only model with standard errors.** WBGT-max variables have been transformed as Z-scores. Controls for twin status, mother's parity, mother's marital status, religion, child's birth month, mother's age in years, survey month, and child's age in months are included in the model but not shown. See Eq. 2 for full model details.

Dependent Variable:	Height-for-age Z-score (HAZ)			
	WBGTmax > 29C			
Model:	(1)	(2)	(3)	(4)
Variables				
Female (child)	0.06***	0.06***	0.06***	0.06***
	(0.010)	(0.010)	(0.010)	(0.010)
Birth place (ref=Clinic)				
Home	-0.04***	-0.04***	-0.04***	-0.04***
	(0.01)	(0.01)	(0.01)	(0.01)
Other	-0.04	-0.04	-0.04	-0.04
	(0.07)	(0.07)	(0.07)	(0.07)
Mother's Education (ref=Higher)				
None	-0.49***	-0.49***	-0.49***	-0.49***
	(0.03)	(0.03)	(0.03)	(0.03)
Primary	-0.41***	-0.41***	-0.41***	-0.41***
	(0.03)	(0.03)	(0.03)	(0.03)
Secondary	-0.24***	-0.24***	-0.24***	-0.24***
	(0.03)	(0.03)	(0.03)	(0.03)
Improved Toilet	0.22***	0.22***	0.22***	0.22***
	(0.01)	(0.01)	(0.01)	(0.01)
Number of extreme days (Z-score)				
Trimester 3	-0.04***	-0.04***	-0.04***	-0.08***
	(0.01)	(0.01)	(0.01)	(0.02)
Trimester 2		0.007	0.006	-0.04**

		(0.01)	(0.01)	(0.02)
Trimester 1			-0.004	-0.04**
			(0.01)	(0.02)
Trimester 0				-0.05***
				(0.02)
Fixed-effects				
DHS cluster	Yes	Yes	Yes	Yes
State-survey year	Yes	Yes	Yes	Yes
Fit statistics				
Observations	198,710	198,710	198,710	198,710
R^2	0.33927	0.33927	0.33927	0.33934
Within R^2	0.01845	0.01846	0.01846	0.01855

Clustered (DHS cluster) standard-errors in parentheses

*Signif. Codes: ***: 0.01, **: 0.05, *: 0.1*

Table S5: **HAZ regression results from non-standardized WBGTmax-only model with standard errors.** Controls for twin status, mother's parity, mother's marital status, religion, child's birth month, mother's age in years, survey month, and child's age in months are included in the model but not shown. See Eq. 2 for full model details.

Dependent Variable:	Height-for-age Z-score (HAZ)			
	WBGTmax > 29C			
Model:	(1)	(2)	(3)	(4)
Variables				
Female (child)	0.06***	0.06***	0.06***	0.06***
	(0.010)	(0.010)	(0.010)	(0.010)
Birth place (ref=Clinic)				
Home	-0.04***	-0.04***	-0.04***	-0.04***
	(0.01)	(0.01)	(0.01)	(0.01)
Other	-0.04	-0.04	-0.04	-0.04
	(0.07)	(0.07)	(0.07)	(0.07)
Mother's Education (ref=Higher)				
None	-0.49***	-0.49***	-0.49***	-0.49***
	(0.03)	(0.03)	(0.03)	(0.03)
Primary	-0.41***	-0.41***	-0.41***	-0.41***
	(0.03)	(0.03)	(0.03)	(0.03)
Secondary	-0.24***	-0.24***	-0.24***	-0.24***
	(0.03)	(0.03)	(0.03)	(0.03)
Improved Toilet	0.22***	0.22***	0.22***	0.22***
	(0.01)	(0.01)	(0.01)	(0.01)
Number of extreme days				
Trimester 3	-0.001***	-0.001***	-0.002***	-0.003***
	(0.0004)	(0.0004)	(0.0005)	(0.0007)
Trimester 2		0.0003	0.0002	-0.001**

		(0.0004)	(0.0004)	(0.0007)
Trimester 1			-0.0001	-0.001**
			(0.0005)	(0.0007)
Trimester 0				-0.002***
				(0.0007)
Fixed-effects				
DHS cluster	Yes	Yes	Yes	Yes
State-survey year	Yes	Yes	Yes	Yes
Fit statistics				
Observations	198,710	198,710	198,710	198,710
R^2	0.33927	0.33927	0.33927	0.33934
Within R^2	0.01845	0.01846	0.01846	0.01855

Clustered (DHS cluster) standard-errors in parentheses

*Signif. Codes: ***: 0.01, **: 0.05, *: 0.1*

Table S6: **HAZ regression results from combined heat model with standard errors.** Tmax and WBGTmax variables have been transformed as Z-scores. Controls for twin status, mother's parity, mother's marital status, religion, child's birth month, mother's age in years, survey month and child's age in months are included in the model but not shown. See Eq. 3 for full model details.

Dependent Variable:	Height-for-age Z-score (HAZ)							
Model:	(1)	(2)	(3)	(4)	(5)	(6)	(7)	(8)
Variables								
Female (child)	0.06***	0.06***	0.06***	0.06***	0.06***	0.06***	0.06***	0.06***
	(0.010)	(0.010)	(0.010)	(0.010)	(0.010)	(0.010)	(0.010)	(0.010)
Birth place (ref=Clinic)								
Home	-0.04***	-0.04***	-0.04***	-0.04***	-0.04***	-0.04***	-0.04***	-0.04***
	(0.01)	(0.01)	(0.01)	(0.01)	(0.01)	(0.01)	(0.01)	(0.01)
Other	-0.04	-0.04	-0.04	-0.04	-0.04	-0.04	-0.04	-0.04
	(0.07)	(0.07)	(0.07)	(0.07)	(0.07)	(0.07)	(0.07)	(0.07)
Mother's Education (ref=Higher)								
None	-0.49***	-0.49***	-0.50***	-0.50***	-0.49***	-0.49***	-0.49***	-0.49***
	(0.03)	(0.03)	(0.03)	(0.03)	(0.03)	(0.03)	(0.03)	(0.03)
Primary	-0.41***	-0.41***	-0.41***	-0.41***	-0.41***	-0.41***	-0.41***	-0.41***
	(0.03)	(0.03)	(0.03)	(0.03)	(0.03)	(0.03)	(0.03)	(0.03)
Secondary	-0.24***	-0.24***	-0.24***	-0.24***	-0.24***	-0.24***	-0.24***	-0.24***
	(0.03)	(0.03)	(0.03)	(0.03)	(0.03)	(0.03)	(0.03)	(0.03)
Improved Toilet	0.22***	0.22***	0.22***	0.22***	0.22***	0.22***	0.22***	0.22***
	(0.01)	(0.01)	(0.01)	(0.01)	(0.01)	(0.01)	(0.01)	(0.01)
Number of extreme days (Z-score)								
Tmax>35C								
Trimester 3	-0.03**	-0.02**	-0.004	-0.02	-0.003	-0.003	-0.002	0.005
	(0.01)	(0.01)	(0.01)	(0.01)	(0.01)	(0.01)	(0.01)	(0.02)
Trimester 2		0.02**	0.03***	0.009	0.01	0.02	0.02	0.02
		(0.01)	(0.01)	(0.01)	(0.01)	(0.01)	(0.01)	(0.01)

Trimester 1			0.03***	0.02	0.01	0.01	0.02	0.02
			(0.01)	(0.01)	(0.01)	(0.01)	(0.01)	(0.02)
Trimester 0				-0.03**	-0.03**	-0.03**	-0.03**	-0.02
				(0.01)	(0.01)	(0.01)	(0.01)	(0.01)
WBGTmax>29C								
Trimester 3					-0.04***	-0.04***	-0.05***	-0.08***
					(0.01)	(0.01)	(0.01)	(0.02)
Trimester 2						-0.01	-0.02	-0.05**
						(0.01)	(0.01)	(0.02)
Trimester 1							-0.02	-0.04**
							(0.01)	(0.02)
Trimester 0								-0.04**
								(0.02)
Fixed-effects								
DHS cluster	Yes	Yes	Yes	Yes	Yes	Yes	Yes	Yes
State-survey year	Yes	Yes	Yes	Yes	Yes	Yes	Yes	Yes
Fit statistics								
Observations	198,710	198,710	198,710	198,710	198,710	198,710	198,710	198,710
R^2	0.33919	0.33923	0.33928	0.33930	0.33939	0.33940	0.33941	0.33944
Within R^2	0.01834	0.01839	0.01846	0.01850	0.01862	0.01864	0.01866	0.01871

Clustered (DHS cluster) standard-errors in parentheses

*Signif. Codes: ***: 0.01, **: 0.05, *: 0.1*

Table S7: **HAZ regression results from non-standardized combined heat model with standard errors.** Controls for twin status, mother's parity, mother's marital status, religion, child's birth month, mother's age in years, survey month and child's age in months are included in the model but not shown. See Eq. 3 for full model details.

Dependent Variable:	Height-for-age Z-score (HAZ)							
Model:	(1)	(2)	(3)	(4)	(5)	(6)	(7)	(8)
Variables								
Female (child)	0.06^{***}	0.06^{***}	0.06^{***}	0.06^{***}	0.06^{***}	0.06^{***}	0.06^{***}	0.06^{***}
	(0.010)	(0.010)	(0.010)	(0.010)	(0.010)	(0.010)	(0.010)	(0.010)
Birth place (ref=Clinic)								
Home	-0.04^{***}	-0.04^{***}	-0.04^{***}	-0.04^{***}	-0.04^{***}	-0.04^{***}	-0.04^{***}	-0.04^{***}
	(0.01)	(0.01)	(0.01)	(0.01)	(0.01)	(0.01)	(0.01)	(0.01)
Other	-0.04	-0.04	-0.04	-0.04	-0.04	-0.04	-0.04	-0.04
	(0.07)	(0.07)	(0.07)	(0.07)	(0.07)	(0.07)	(0.07)	(0.07)
Mother's Education (ref=Higher)								
None	-0.49^{***}	-0.49^{***}	-0.50^{***}	-0.50^{***}	-0.49^{***}	-0.49^{***}	-0.49^{***}	-0.49^{***}
	(0.03)	(0.03)	(0.03)	(0.03)	(0.03)	(0.03)	(0.03)	(0.03)
Primary	-0.41^{***}	-0.41^{***}	-0.41^{***}	-0.41^{***}	-0.41^{***}	-0.41^{***}	-0.41^{***}	-0.41^{***}
	(0.03)	(0.03)	(0.03)	(0.03)	(0.03)	(0.03)	(0.03)	(0.03)
Secondary	-0.24^{***}	-0.24^{***}	-0.24^{***}	-0.24^{***}	-0.24^{***}	-0.24^{***}	-0.24^{***}	-0.24^{***}
	(0.03)	(0.03)	(0.03)	(0.03)	(0.03)	(0.03)	(0.03)	(0.03)
Improved Toilet	0.22^{***}	0.22^{***}	0.22^{***}	0.22^{***}	0.22^{***}	0.22^{***}	0.22^{***}	0.22^{***}
	(0.01)	(0.01)	(0.01)	(0.01)	(0.01)	(0.01)	(0.01)	(0.01)
Number of extreme days								
Tmax>35C								
Trimester 3	-0.0009^{**}	-0.0008^{**}	-0.0001	-0.0007	-0.0001	-0.0001	-6.5×10^{-5}	0.0002
	(0.0004)	(0.0004)	(0.0005)	(0.0005)	(0.0006)	(0.0006)	(0.0006)	(0.0006)
Trimester 2		0.0008^{**}	0.001^{***}	0.0003	0.0004	0.0006	0.0007	0.0009
		(0.0004)	(0.0004)	(0.0005)	(0.0005)	(0.0006)	(0.0006)	(0.0006)

Trimester 1			0.001***	0.0007	0.0005	0.0005	0.0007	0.0009
			(0.0005)	(0.0006)	(0.0006)	(0.0006)	(0.0006)	(0.0006)
Trimester 0				-0.001**	-0.001**	-0.001**	-0.001**	-0.0008
				(0.0006)	(0.0006)	(0.0006)	(0.0006)	(0.0006)
WBGTmax>29C								
Trimester 3					-0.001***	-0.001***	-0.002***	-0.003***
					(0.0004)	(0.0004)	(0.0005)	(0.0007)
Trimester 2						-0.0005	-0.0007	-0.002**
						(0.0004)	(0.0005)	(0.0007)
Trimester 1							-0.0007	-0.002**
							(0.0006)	(0.0007)
Trimester 0								-0.002**
								(0.0007)
Fixed-effects								
DHS cluster	Yes	Yes	Yes	Yes	Yes	Yes	Yes	Yes
State-survey year	Yes	Yes	Yes	Yes	Yes	Yes	Yes	Yes
Fit statistics								
Observations	198,710	198,710	198,710	198,710	198,710	198,710	198,710	198,710
R^2	0.33919	0.33923	0.33928	0.33930	0.33939	0.33940	0.33941	0.33944
Within R^2	0.01834	0.01839	0.01846	0.01850	0.01862	0.01864	0.01866	0.01871

Clustered (DHS cluster) standard-errors in parentheses

*Signif. Codes: ***: 0.01, **: 0.05, *: 0.1*

Table S8: **HAZ regression results from Tmax-only model with household fixed effects.** Controls for twin status, mother's parity, mother's marital status, religion, child's birth month, mother's age in years, survey month, and child's age in months are included in the model but not shown.

Dependent Variable:	Height-for-age Z-score (HAZ)			
Model:	(1)	(2)	(3)	(4)
	Tmax > 35C			
Variables				
Female (child)	0.06***	0.06***	0.06***	0.06***
	(0.02)	(0.02)	(0.02)	(0.02)
Birth place (ref=Clinic)				
Home	0.04	0.04	0.04	0.04
	(0.04)	(0.04)	(0.04)	(0.04)
Other	0.07	0.07	0.07	0.07
	(0.16)	(0.16)	(0.16)	(0.16)
Mother's Education (ref=Higher)				
None	-0.33**	-0.33**	-0.33**	-0.33**
	(0.16)	(0.16)	(0.16)	(0.16)
Primary	-0.30*	-0.30*	-0.30*	-0.30*
	(0.18)	(0.18)	(0.18)	(0.18)
Secondary	-0.15	-0.15	-0.15	-0.15
	(0.14)	(0.14)	(0.14)	(0.14)
Improved Toilet	0.29**	0.29**	0.29**	0.29**
	(0.14)	(0.14)	(0.14)	(0.14)
Number of extreme days				
Trimester 3	-0.0007	-0.0007	3.3×10^{-5}	-0.0009
	(0.0008)	(0.0008)	(0.001)	(0.001)
Trimester 2		0.0001	0.0003	-0.0009
		(0.0008)	(0.0008)	(0.001)

Trimester 1			0.001	0.0004
			(0.001)	(0.001)
Trimester 0				-0.002*
				(0.001)
Fixed-effects				
Household	Yes	Yes	Yes	Yes
State-survey year	Yes	Yes	Yes	Yes
Fit statistics				
Observations	91,529	91,529	91,529	91,529
R^2	0.67106	0.67106	0.67109	0.67116
Within R^2	0.02015	0.02015	0.02025	0.02046

Clustered (DHS cluster) standard-errors in parentheses

*Signif. Codes: ***: 0.01, **: 0.05, *: 0.1*

Table S9: **HAZ regression results from WBGTmax-only model with household fixed effects.** Controls for twin status, mother's parity, mother's marital status, religion, child's birth month, mother's age in years, survey month, and child's age in months are included in the model but not shown.

Dependent Variable:	Height-for-age Z-score (HAZ)			
	WBGTmax > 29C			
Model:	(1)	(2)	(3)	(4)
Variables				
Female (child)	0.06***	0.06***	0.06***	0.06***
	(0.02)	(0.02)	(0.02)	(0.02)
Birth place (ref=Clinic)				
Home	0.04	0.04	0.04	0.04
	(0.04)	(0.04)	(0.04)	(0.04)
Other	0.07	0.07	0.07	0.07
	(0.15)	(0.15)	(0.15)	(0.16)
Mother's Education (ref=Higher)				
None	-0.33**	-0.33**	-0.33**	-0.33**
	(0.16)	(0.16)	(0.16)	(0.16)
Primary	-0.30*	-0.30*	-0.30*	-0.30*
	(0.18)	(0.18)	(0.18)	(0.18)
Secondary	-0.15	-0.15	-0.15	-0.15
	(0.14)	(0.14)	(0.14)	(0.14)
Improved Toilet	0.29**	0.29**	0.29**	0.30**
	(0.14)	(0.14)	(0.14)	(0.14)
Number of extreme days				
Trimester 3	-0.0006	-0.0007	-5×10^{-5}	-0.002
	(0.0008)	(0.0008)	(0.001)	(0.001)
Trimester 2		-0.0003	-8.2×10^{-5}	-0.002

		(0.0008)	(0.0008)	(0.001)
Trimester 1			0.0009	-0.0006
			(0.001)	(0.001)
Trimester 0				-0.003*
				(0.001)
Fixed-effects				
Household	Yes	Yes	Yes	Yes
State-survey year	Yes	Yes	Yes	Yes
Fit statistics				
Observations	91,529	91,529	91,529	91,529
R^2	0.67106	0.67106	0.67108	0.67116
Within R^2	0.02015	0.02016	0.02021	0.02045

Clustered (DHS cluster) standard-errors in parentheses

*Signif. Codes: ***: 0.01, **: 0.05, *: 0.1*

Table S10: **Supplementary stunting regression results from Tmax-only model with standard errors.** Controls for twin status, mother's parity, mother's marital status, religion, child's birth month, mother's age in years, survey month, and child's age in months are included in the model but not shown. See Eq. 2 for full model details. In the case of stunting, which is a binary outcome, Eq. 2 is a linear probability model.

Dependent Variable:	Stunting			
	Tmax > 35C			
Model:	(1)	(2)	(3)	(4)
Variables				
Female (child)	-0.01***	-0.01***	-0.01***	-0.01***
	(0.003)	(0.003)	(0.003)	(0.003)
Birth place (ref=Clinic)				
Home	0.008*	0.008*	0.008*	0.008*
	(0.004)	(0.004)	(0.004)	(0.004)
Other	0.02	0.02	0.02	0.02
	(0.03)	(0.03)	(0.03)	(0.03)
Mother's Education (ref=Higher)				
None	0.14***	0.14***	0.14***	0.14***
	(0.008)	(0.008)	(0.008)	(0.008)
Primary	0.11***	0.11***	0.11***	0.11***
	(0.008)	(0.008)	(0.008)	(0.008)
Secondary	0.06***	0.06***	0.06***	0.06***
	(0.007)	(0.007)	(0.007)	(0.007)
Improved Toilet	-0.06***	-0.06***	-0.06***	-0.06***
	(0.005)	(0.005)	(0.005)	(0.005)
Number of extreme days				
Trimester 3	0.0002**	0.0002*	6.6×10^{-5}	8.5×10^{-5}
	(0.0001)	(0.0001)	(0.0001)	(0.0002)

Trimester 2		-0.0002*	-0.0003**	-0.0002
		(0.0001)	(0.0001)	(0.0002)
Trimester 1			-0.0003*	-0.0003
			(0.0001)	(0.0002)
Trimester 0				3.7×10^{-5}
				(0.0002)
Fixed-effects				
DHS cluster	Yes	Yes	Yes	Yes
State-survey year	Yes	Yes	Yes	Yes
Fit statistics				
Observations	198,710	198,710	198,710	198,710
R^2	0.27427	0.27429	0.27432	0.27432
Within R^2	0.01566	0.01569	0.01572	0.01572

Clustered (DHS cluster) standard-errors in parentheses

*Signif. Codes: ***: 0.01, **: 0.05, *: 0.1*

Table S11: **Supplementary stunting regression results from WBGTmax-only model with standard errors.** Controls for twin status, mother's parity, mother's marital status, religion, child's birth month, mother's age in years, survey month, and child's age in months are included in the model but not shown. See Eq. 2 for full model details. In the case of stunting, which is a binary outcome, Eq. 2 is a linear probability model.

Dependent Variable:	Stunting			
	WBGTmax > 29C			
Model:	(1)	(2)	(3)	(4)
Variables				
Female (child)	-0.01***	-0.01***	-0.01***	-0.01***
	(0.003)	(0.003)	(0.003)	(0.003)
Birth place (ref=Clinic)				
Home	0.008*	0.008*	0.008*	0.008*
	(0.004)	(0.004)	(0.004)	(0.004)
Other	0.02	0.02	0.02	0.02
	(0.03)	(0.03)	(0.03)	(0.03)
Mother's Education (ref=Higher)				
None	0.14***	0.14***	0.14***	0.14***
	(0.008)	(0.008)	(0.008)	(0.008)
Primary	0.11***	0.11***	0.11***	0.11***
	(0.008)	(0.008)	(0.008)	(0.008)
Secondary	0.06***	0.06***	0.06***	0.06***
	(0.007)	(0.007)	(0.007)	(0.007)
Improved Toilet	-0.06***	-0.06***	-0.06***	-0.06***
	(0.005)	(0.005)	(0.005)	(0.005)
Number of extreme days				
Trimester 3	0.0003***	0.0003***	0.0003**	0.0006***
	(0.0001)	(0.0001)	(0.0001)	(0.0002)

Trimester 2		-0.0001	-0.0001	0.0002
		(0.0001)	(0.0001)	(0.0002)
Trimester 1			1.3×10^{-5}	0.0003
			(0.0001)	(0.0002)
Trimester 0				0.0004*
				(0.0002)
Fixed-effects				
DHS cluster	Yes	Yes	Yes	Yes
State-survey year	Yes	Yes	Yes	Yes
Fit statistics				
Observations	198,710	198,710	198,710	198,710
R^2	0.27432	0.27434	0.27434	0.27437
Within R^2	0.01573	0.01575	0.01575	0.01579

Clustered (DHS cluster) standard-errors in parentheses

*Signif. Codes: ***: 0.01, **: 0.05, *: 0.1*

Table S12: **Stunting regression results from combined heat model with standard errors.** Controls for twin status, mother's parity, mother's marital status, religion, child's birth month, mother's age in years, survey month and child's age in months are included in the model but not shown. See Eq. 3 for full model details. In the case of stunting, which is a binary outcome, Eq. 3 is a linear probability model.

Dependent Variable:	Stunting							
Model:	(1)	(2)	(3)	(4)	(5)	(6)	(7)	(8)
Variables								
Female (child)	-0.01***	-0.01***	-0.01***	-0.01***	-0.01***	-0.01***	-0.01***	-0.01***
	(0.003)	(0.003)	(0.003)	(0.003)	(0.003)	(0.003)	(0.003)	(0.003)
Birth place (ref=Clinic)								
Home	0.008*	0.008*	0.008*	0.008*	0.008*	0.008*	0.008*	0.008*
	(0.004)	(0.004)	(0.004)	(0.004)	(0.004)	(0.004)	(0.004)	(0.004)
Other	0.02	0.02	0.02	0.02	0.02	0.02	0.02	0.02
	(0.03)	(0.03)	(0.03)	(0.03)	(0.03)	(0.03)	(0.03)	(0.03)
Mother's Education (ref=Higher)								
None	0.14***	0.14***	0.14***	0.14***	0.14***	0.14***	0.14***	0.14***
	(0.008)	(0.008)	(0.008)	(0.008)	(0.008)	(0.008)	(0.008)	(0.008)
Primary	0.11***	0.11***	0.11***	0.11***	0.11***	0.11***	0.11***	0.11***
	(0.008)	(0.008)	(0.008)	(0.008)	(0.008)	(0.008)	(0.008)	(0.008)
Secondary	0.06***	0.06***	0.06***	0.06***	0.06***	0.06***	0.06***	0.06***
	(0.007)	(0.007)	(0.007)	(0.007)	(0.007)	(0.007)	(0.007)	(0.007)
Improved Toilet	-0.06***	-0.06***	-0.06***	-0.06***	-0.06***	-0.06***	-0.06***	-0.06***
	(0.005)	(0.005)	(0.005)	(0.005)	(0.005)	(0.005)	(0.005)	(0.005)
Number of extreme days								
Tmax>35C								
Trimester 3	0.0002**	0.0002*	6.6×10^{-5}	8.5×10^{-5}	-6.1×10^{-5}	-6.1×10^{-5}	-7×10^{-5}	-0.0001
	(0.0001)	(0.0001)	(0.0001)	(0.0002)	(0.0002)	(0.0002)	(0.0002)	(0.0002)
Trimester 2		-0.0002*	-0.0003**	-0.0002	-0.0002	-0.0002	-0.0002	-0.0003*

		(0.0001)	(0.0001)	(0.0002)	(0.0002)	(0.0002)	(0.0002)	(0.0002)
Trimester 1			-0.0003*	-0.0003	-0.0002	-0.0002	-0.0002	-0.0003
			(0.0001)	(0.0002)	(0.0002)	(0.0002)	(0.0002)	(0.0002)
Trimester 0				3.7×10^{-5}	5.6×10^{-5}	5.4×10^{-5}	5.6×10^{-5}	-7.2×10^{-5}
				(0.0002)	(0.0002)	(0.0002)	(0.0002)	(0.0002)
WBGTmax>29C								
Trimester 3					0.0003***	0.0003**	0.0004**	0.0007***
					(0.0001)	(0.0001)	(0.0002)	(0.0002)
Trimester 2						-1.5×10^{-5}	1.2×10^{-5}	0.0003
						(0.0001)	(0.0001)	(0.0002)
Trimester 1							0.0001	0.0004*
							(0.0002)	(0.0002)
Trimester 0								0.0004*
								(0.0002)
Fixed-effects								
DHS cluster	Yes	Yes	Yes	Yes	Yes	Yes	Yes	Yes
State-survey year	Yes	Yes	Yes	Yes	Yes	Yes	Yes	Yes
Fit statistics								
Observations	198,710	198,710	198,710	198,710	198,710	198,710	198,710	198,710
R^2	0.27427	0.27429	0.27432	0.27432	0.27437	0.27437	0.27438	0.27440
Within R^2	0.01566	0.01569	0.01572	0.01572	0.01580	0.01580	0.01580	0.01584

Clustered (DHS cluster) standard-errors in parentheses

*Signif. Codes: ***: 0.01, **: 0.05, *: 0.1*
